# CRISPR screens and quantitative proteomics reveal remodeling of the aryl hydrocarbon receptor–driven proteome through PARP7 activity

**DOI:** 10.1073/pnas.2424985122

**Published:** 2025-06-10

**Authors:** Andrii Gorelik, Joao A. Paulo, Christina B. Schroeter, Melanie Lad, Abigail Shurr, Chara Mastrokalou, Samrah Siddiqi, Osamu Suyari, John Brognard, David Walter, Jason Matthews, Timothy M. Palmer, Steven P. Gygi, Ivan Ahel

**Affiliations:** ^a^Sir William Dunn School of Pathology, University of Oxford, Oxford OX1 3RE, United Kingdom; ^b^Department of Cell Biology, Harvard Medical School, Boston, MA 02115; ^c^Department of Neurology, Medical Faculty and University Hospital Düsseldorf, Heinrich Heine University, Düsseldorf 40225, Germany; ^d^Cancer Research Horizons, Joint AstraZeneca-Cancer Research Horizons Functional Genomics Centre, Cambridge CB2 0AW, United Kingdom; ^e^Laboratory of Cell and Developmental Signaling, Center for Cancer Research, National Cancer Institute, Frederick, MD 21702; ^f^Department of Nutrition, Institute of Basic Medical Sciences, University of Oslo, Oslo 0317, Norway; ^g^Department of Pharmacology and Toxicology, University of Toronto, Toronto, ON M5S 1A8, Canada; ^h^Biomedical Institute for Multimorbidity, Centre for Biomedicine, Hull York Medical School, University of Hull, Hull HU6 7RX, United Kingdom

**Keywords:** aryl hydrocarbon receptor, ADP-ribosylation, PARP, CRISPR, proteomics

## Abstract

PARP7 is an enzyme that uses donor substrate NAD^+^ to attach a single ADP-ribose moiety onto proteins related to immunity, transcription, and cell growth and motility. Despite the importance of PARP7 in these processes, PARP7 signaling networks remain underresearched. Here, we used genome-wide CRISPR screens and multiplex quantitative proteomics in distinct lung cancer cell lines treated with a PARP7 inhibitor to better understand PARP7 molecular functions. We find that manipulating the aryl hydrocarbon receptor (AHR) transcriptional activity mediates PARP7 inhibitor sensitivity and triggers robust changes to the AHR-controlled proteome (AHR-ome). One of the striking features of such AHR-ome remodeling was the downregulation of filamins A and B concurrent with the induction of the corresponding E3 ubiquitin ligase ASB2. We also show that suppressor of cytokine signaling 3 (SOCS3) crosstalks to AHR. Inhibition of PARP7 in SOCS3 knockout cells leads to reduced viability compared to wild-type cells treated with a PARP7 inhibitor. Our results reveal signaling interplay between PARP7, AHR, and SOCS3 and establish an invaluable resource to study the role of PARP7 in the regulation of AHR signaling and innate immunity through its ADP-ribosyl transferase activity.

## Significance

Mono-ADP-ribosyltransferase PARP7 has emerged as a promising therapeutic target for the lung, prostate, and ovarian cancer. However, the mechanisms of PARP7 signaling remain elusive. In this study, we demonstrate that PARP7 enzymatic activity coordinates with basal aryl hydrocarbon receptor (AHR) transcriptional activity to mediate homeostasis in lung cancer cells. Disruption of this crosstalk through simultaneous PARP7 inhibition and AHR activation leads to extensive remodeling of the AHR-driven proteome and enhanced cancer cell growth suppression. Mechanistically, we show that concurrent PARP7 inhibition and AHR activation lead to ASB2 E3 ligase induction, which negatively regulates the levels of important actin-binding proteins filamin A and B. Additionally, we establish that SOCS3-null cells are more sensitive to PARP7 inhibition, highlighting a promising synthetic lethal interaction.

ADP-ribosylation (ADPr) of proteins is a highly conserved, therapeutically relevant posttranslational modification (PTM) that has been linked to critical processes such as DNA damage, pathogen infections, and immune responses (1, 2). One of the enzyme families that catalyzes this PTM in vertebrates is the PARP enzymes, which add ADP-ribose onto specific protein targets using the donor substrate NAD^+^ (3–5). The dynamic nature of this modification is achieved via several ADP-ribosyl hydrolases that reverse ADPr (6).

The best-understood PARPs, PARP1/2, are involved in DNA repair; however, less is known about PARPs with roles in immunity and inflammation such as PARP7 (7–10). PARP7 is a nuclear PARP that contains a C-terminal catalytic domain with mono-ADPr activity, a CCCH-type zinc finger and a PAR-binding WWE motif, with the latter two being important for PARP7 nuclear localization (11). Although viable, PARP7-null mice exhibit defects such as hemorrhaging and microaneurysms, while female PARP7-null mice are infertile (12). Additionally, PARP7 has important roles in regulating embryonic stem cell pluripotency (13).

Initially, PARP7 was named 2,3,7,8-tetrachlorodibenzo-p-dioxin (TCDD)-inducible poly-ADP-ribose polymerase (TIPARP) due to its close interplay with the transcription factor, aryl hydrocarbon receptor (AHR), and induction of PARP7 expression through AHR agonists TCDD, benzo-a-pyrene, 6-formylindolo(3,2-b) carbazole, kynurenine, and others (11, 14). Adding to the crosstalk between AHR and PARP7, the latter ADP-ribosylates AHR but the function of this modification remains unclear (15). Apart from AHR, several receptors have been proposed to regulate PARP7 and reciprocally be regulated by it. Examples include the androgen receptor (AR), liver X receptor (LXR), and estrogen receptor alpha (ERα) (16–18). PARP7 has been proposed to ADP-ribosylate cysteine and glutamate residues on target proteins which often occur on proteins related to RNA processes and immunity (19, 20). AR is ADP-ribosylated by PARP7 on multiple cysteine residues which modulate AR transcriptional activity via a PARP9/DTX3L recruitment complex that may act as a reader of Cys-ADPr (21). So far, human Cys-ADPr hydrolases have not been discovered (22). In addition to its Cys-ADPr activity, PARP7 also ADP-ribosylates glutamate residues of proteins such as α-tubulin which promotes microtubule instability in ovarian cancer (20).

In recent years, many PARP inhibitors have been developed that target not just the DNA-damage-associated PARP1/2 enzymes but other key PARP-family members. Among these, PARP7 has recently emerged as a promising therapeutic target in various cancers, such as lung and prostate and is potently inhibited by a selective inhibitor RBN2397 (23, 24). It has been shown that PARP7 inhibition by RBN2397 induces the IFN response of lung cancer cells through the activation of nucleic acid sensing via the cGAS/STING pathway and the downstream Janus kinase/signal transducer and activator of transcription (JAK/STAT) signaling pathway (24).

The PARP7 inhibitor (PARP7i), RBN2397, has been used previously in a whole-genome CRISPR screen of H1373 cells to show that the loss of either AHR or PARP7 confers resistance to PARP7 inhibition (24, 25). However, a comprehensive search for other PARP7i resistance genes in different cell lines has not been attempted. Moreover, genetic interactions that drive PARP7i sensitivity have not been uncovered. It is still not known how simultaneous AHR and PARP7 manipulation affects proteome-wide signaling.

Here, we performed a whole-genome CRISPR screen in combination with PARP7 inhibition to identify PARP7i resistance and sensitivity factors in a panel of three distinct lung cancer cell lines. We find that not only the loss of AHR but also the deletion of its binding partners AIP (AHR interacting protein) and translocator ARNT (nuclear translocator) may contribute to RBN2397 resistance. In addition to AHR-specific hits, we identify MAPK14 (p38α), BMPR1A, LCMT1, and PPP2R2A as potential drivers of PARP7i resistance. Quantitative proteomics of AHR agonist- and antagonist-treated cells revealed robust remodeling of the proteome. Notably, the AHR agonist tapinarof in combination with RBN2397 can result in a dramatic increase in levels of the AHR target ASB2 which triggered the downregulation of its substrates filamins A and B, structural proteins important for cell proliferation and motility. Together with the induction of the cell cycle arrest proteins p21 (CDKN1A) and p27 (CDKN1B), this underpins the pronounced decrease in cancer cell viability via the simultaneous AHR activation and PARP7 inhibition. Strikingly, among the combined several hundred potential sensitivity hits, AHR target gene, *SOCS3*, emerged as the only hit shared by all three cell lines. SOCS3-deficient cells were more sensitive to PARP7 inhibition, concurrent with the induction of IFN- and AHR-induced proteins, thus establishing a reciprocal regulation between AHR and SOCS3 mediated by PARP7 activity.

Together, our findings highlight the crucial role of PARP7 in AHR signaling and innate immune responses through its ADP-ribosyl transferase activity, providing a valuable resource for the future exploration of molecular mechanisms of PARP7 signaling.

## Results

### A Genome-Wide Synthetic Lethality CRISPR Screen Identifies Multiple Resistance Hits upon PARP7 Inhibition.

**Fig. 1.**
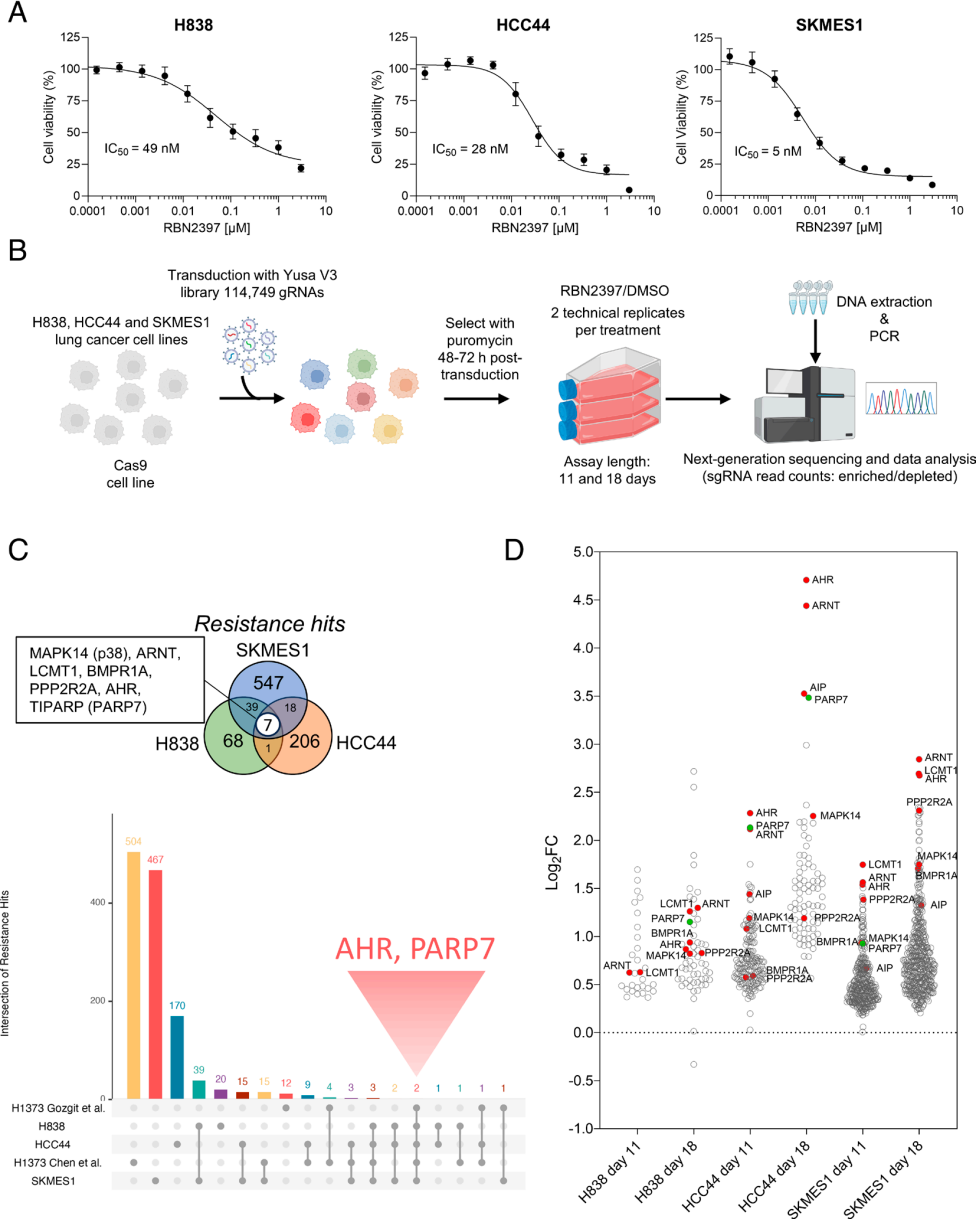
A genome-wide CRISPR synthetic lethality screen with a panel of lung cancer cell lines identifies PARP7 inhibitor resistance hits. (*A*) Cell viability of selected cell lines treated with a range of concentrations of RBN2397 for 6 d measured in a CellTiter-Glo® assay. Data are shown as mean ± SEM of n = 3 biological replicates. (*B*) Schematic representation of the CRISPR screen workflow. (*C*) Overlap of resistance hits identified from the screen and compared to published studies (Dataset S4). The union of significant hits across the two timepoints (day 11 and 18) was used for each cell line of the screen. (*D*) Resistance hits identified from the genome-wide CRISPR screen in HCC44, SKMES1, and H838 cells with FDR < 0.1, shown as log_2_ fold-change of RBN2397 treatment relative to DMSO. PARP7 (TIPARP) is shown in green.

Despite active PARP7 inhibitor development, the regulators of PARP7 inhibitor resistance are not well understood. We aimed to uncover PARP7i resistance mechanisms by focusing on lung cancer cell models as strong PARP7 dependency has been shown previously in this cancer type (26). To accomplish this, we chose three lung cancer cell lines: squamous cell carcinoma, SKMES1, and two adenocarcinoma cell lines, HCC44 and H838, which all have high PARP7 expression and PARP7 dependency relative to other lung cancer cell lines according to Cancer Dependency Map (DepMap https://depmap.org/). We first measured the viability of these cell lines across a range of RBN2397 concentrations. All three cell lines were sensitive to PARP7 inhibition by RBN2397 with IC_50_ values in the low nanomolar range (Fig. 1*A*). We then performed a whole-genome CRISPR screen utilizing the Yusa V3 gRNA library in combination with RBN2397 at a concentration of 900 nM to achieve complete PARP7 inhibition (Fig. 1*B* and Datasets S1–S3). Western blotting using an ADP-ribose-specific antibody revealed minimal effects of PARP7 inhibition on total ADPr levels, whereas a PARP1/2 inhibitor olaparib decreased total ADPr (*SI Appendix*, Fig. S1). This suggests that using relatively high concentrations of PARP7 inhibitor does not substantially affect PARP1/2-mediated ADPr.

In total, we detected 821 enriched genes across HCC44, SKMES1, and H838 cell lines (Fig. 1*C* and *SI Appendix*, Figs. S2–S7). The screen yielded seven resistance hits (AHR, ARNT, PARP7, MAPK14 (p38α), LCMT1, BMPR1A, and PPP2R2A) shared by the three cell lines. Notably, AHR loss has previously been associated with PARP7i resistance in H1373 cells and ARNT is a binding partner of AHR which is required for its transcriptional activity (Dataset S4) (25, 27). Loss of AHR interacting protein (AIP) may also drive resistance in HCC44 and SKMES1 cells (Fig. 1*D*). Interestingly, LCMT1 and PPP2R2A exhibit the highest positive codependency correlation according to DepMap and both were identified previously as PARP7i resistance hits in an independent study (Dataset S4) (25, 26, 28). LCMT1 methylates the C-terminal leucine of PPP2R2A to regulate PP2A substrate specificity and has been implicated in prostate cancer through AR regulation (29, 30). The other hit, BMPR1A, is a type IA receptor for TGF-beta superfamily ligands that has been linked to Wnt signaling (31). Among its diverse roles, the p38 kinase regulates STAT1 signaling. A pan-PARP inhibitor thioparib induces phosphorylation of STAT1 on Tyr701, required for its full transcriptional activity, which is dependent on p38 activity (32). Thus, the identified resistance hits may play important roles in PARP7-mediated signaling with AHR signaling being the top pathway that responds to PARP7 inhibition.

### PARP7 Inhibition Cooperates with AHR Activation to Remodel the Proteome and Regulate Filamin A and B Levels.

**Fig. 2.**
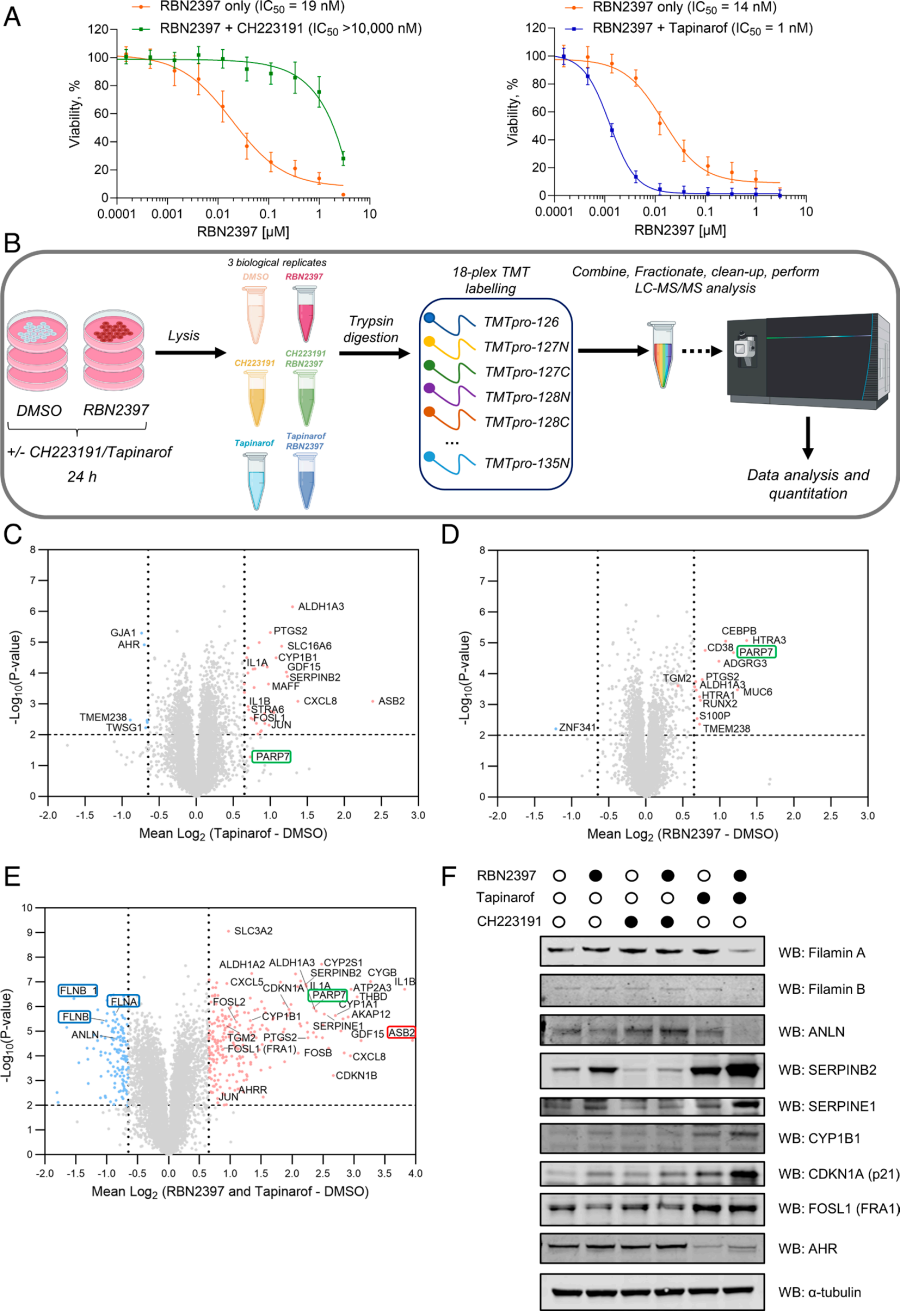
Whole-proteome changes induced by PARP7 inhibition and AHR agonist and antagonist. (*A*) Cell viability of HCC44 cells treated with a range of concentrations of RBN2397 with or without 1 μM CH223191 or 1 μM tapinarof for 6 d, measured in a CellTiter-Glo^®^ assay. Data are shown as mean ± SEM of n = 3 biological replicates (2 technical replicates each). (*B*) TMT 18-plex quantitative proteomics workflow. (*C*–*E*) Quantitative proteomics (TMT 18-plex) volcano plots showing significantly upregulated (red) and downregulated (blue) proteins in HCC44 cells treated with 1 μM tapinarof, 1 μM RBN2397, and a combination of RBN2397 and tapinarof (both at 1 μM), respectively. (*F*) Western blot validation of up- and downregulated hits from proteomics experiments in panels *C*–*E* in HCC44 cells.

Having shown that AHR is one of the key determinants of PARP7i resistance we sought to validate this in a viability assay with HCC44 cells by utilizing AHR antagonist CH223191. Consistent with the CRISPR screen findings (Fig. 1 *C* and *D*), we observed that AHR inactivation with CH223191 rescued the RBN2397-induced reduction in cell viability (Fig. 2*A*). On the contrary, AHR agonist tapinarof in combination with RBN2397 greatly reduced cell viability (Fig. 2*A*). Tapinarof and CH223191 alone did not substantially affect the viability of HCC44 cells up to a concentration of 6 µM (*SI Appendix*, Fig. S8). We further validated the effects of RBN2397 combination with either CH223191 or tapinarof in SKMES1 and H838 cell lines (*SI Appendix*, Fig. S9). Similarly to HCC44 cells, SKMES1 and H838 were sensitized or made resistant to RBN2397 when AHR was respectively activated or inactivated.

We next sought to investigate the proteome-wide changes linked to AHR signaling that cause the above phenotypes. Although PARP7 inhibition is known to promote the expression of proteins linked to AHR signaling, comprehensive proteome-wide profiling treated with PARP7i in combination with AHR (ant)agonist has not been previously reported (24). To address this, we profiled HCC44 cells with PARP7 inhibitor alone or in combination with AHR (ant)agonist using 18-plex-TMT proteomics (Fig. 2*B*). In total, we identified and quantified over 8600 proteins (Dataset S5).

To examine the consequences of AHR activation on the proteome of the HCC44 cell line, we treated these cells with AHR agonist tapinarof. AHR activation upregulated multiple known AHR targets such as ASB2, CYP1B1, IL1B, ALDH1A3, and SERPINB2 (Fig. 2*C*). Consistent with AHR activation-induced degradation we noted a decrease in AHR levels (Fig. 2*C*).

Inhibition of PARP7 with RBN2397 elevated the levels of multiple proteins, including PARP7 itself (Fig. 2*D*) but did not affect the levels of other PARPs (Dataset S5). Some of the induced proteins, such as ALDH1A3 and PTGS2 were also upregulated in the tapinarof-treated cells. To investigate whether the upregulation of this subset of proteins was through AHR, we performed a simultaneous treatment with both RBN2397 and AHR antagonist CH223191 (*SI Appendix*, Fig. S10). Interestingly, this treatment decreased the levels of most of RBN2397-induced proteins. However, PARP7 levels remained high relative to the DMSO control, suggesting that PARP7 expression is not exclusively controlled by AHR.

We then treated HCC44 cells with a combination of tapinarof and RBN2397 and observed extensive remodeling of the proteome with hundreds of proteins being up- or downregulated (Fig. 2*E*). Notably, IL1B was further upregulated which is consistent with published literature on IL1B as an AHR target (27). We observed upregulation of other AHR target genes such as CYP1A1 and CYP1B1, SERPINE1 and SERPINB2 (Fig. 2*E*). The reduced cell viability of RBN2397-treated cancer cell lines has been linked to senescence previously (24) and senescence markers CDKN1A (p21) and CKDN1B (p27) were also dramatically increased upon this treatment (Fig. 2*E*). Using western blotting, we successfully validated that CYP1B1, SERPINE1, SERPINB2, and CDKN1A (p21) proteins are upregulated upon simultaneous AHR activation and PARP7 inhibition (Fig. 2*F*). In case of SERPINB2, PARP7 inhibition increased its levels which could be reversed through CH223191 treatment.

AP1 complex components FOSL1 (FRA1), FOSB, and JUN were all upregulated (Fig. 2 *E* and *F*). Recently, PARP7 inhibition was shown to promote the proteasomal degradation of the transcription factor FRA1 and induce apoptosis, with cells simultaneously expressing high levels of FRA1 and PARP7 being most sensitive to PARP7i (33). Both AHR activation through tapinarof treatment and a combined treatment of cells with tapinarof and RBN2397 increased FRA1 levels and this was validated by western blot (Fig. 2 *E* and *F*). Our data suggest that FRA1 is upregulated through AHR activation which may contribute to reduced cancer cell viability.

Strikingly, the most upregulated protein in the tapinarof/RBN2397 condition was ASB2 (Fig. 2*E*). ASB2 is an E3 ubiquitin ligase known to target filamins A and B for proteasomal degradation (34). Gratifyingly, filamin A and B (but not filamin C) levels were reduced in this condition (Fig. 2*E*). While tapinarof alone induced a six-fold upregulation of ASB2 without affecting filamin levels, the combination with RBN2397 triggered a 16-fold increase, which correlated with substantial filamin A/B degradation. The greater increase in ASB2 appears to trigger filamin A/B degradation. Decrease in filamin A and B levels after 24 h of combined tapinarof/RBN2397 treatment was confirmed by western blot analysis (Fig. 2*F*). Prolonged tapinarof/RBN2397 treatment (72 h) led to ASB2 upregulation and a near-complete filamin A degradation (*SI Appendix*, Fig. S11). Interestingly, when we performed the same treatment in SKMES1 and H838 cells, neither proteomics nor immunoblotting revealed filamin degradation (*SI Appendix*, Figs. S12–S14). However, another actin-binding protein, anillin (ANLN), was consistently downregulated across all three cell lines (Fig. 2 *E* and *F* and *SI Appendix*, Figs. S12–S14 and Dataset S5). Additionally, we noted that p21 was increased in SKMES1 and H838 cell lines similarly to HCC44. We observed that AHR targets SERPINE1 and SERPINB2 were not substantially increased in H838 cells suggesting comparatively lower basal AHR activity in this cell line. Complementary phosphoproteomic analyses across the three cell lines further substantiated many of the observed signaling differences (Dataset S6).

Overall, we have shown that PARP7 and AHR cooperate to modulate multiple signaling networks. In particular, the downregulation of filamin A/B protein levels upon PARP7 inhibition and AHR activation may contribute to the reduced proliferative capacity of HCC44 cells.

### SOCS3 Knockout Sensitizes HCC44 Cells to PARP7 Inhibition.

**Fig. 3.**
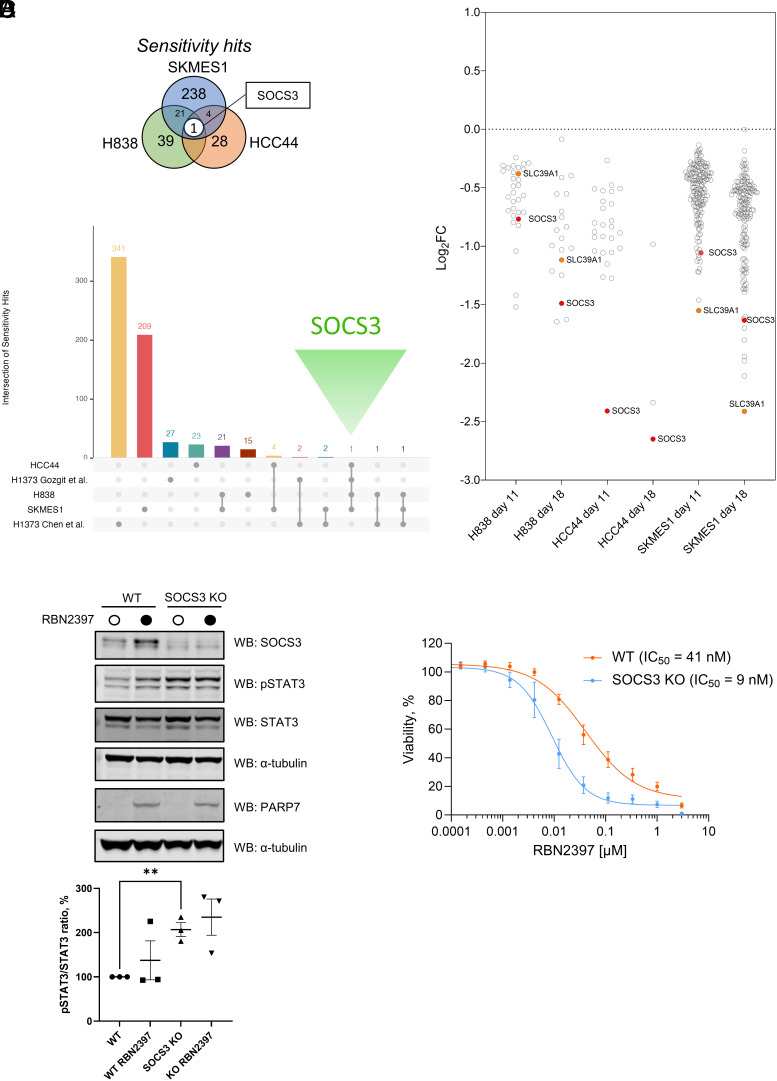
A genome-wide CRISPR synthetic lethality screen identifies SOCS3 as a conserved PARP7 inhibitor sensitivity hit. (*A*) Overlap of sensitivity hits identified from the screen and compared to published studies (Dataset S4). The union of significant hits across the two timepoints (day 11 and 18) was used for each cell line of the screen. (*B*) Sensitivity hits identified from the genome-wide CRISPR screen in HCC44, SKMES1, and H838 cells with FDR < 0.1, shown as log_2_ fold-change of RBN2397 treatment relative to DMSO. (*C*) Western blotting analysis of SOCS3 knockout HCC44 cell lines compared to wild-type, treated with or without 1 µM RBN2397. STAT3 phosphorylation was detected with a phospho-STAT3-specific antibody. Western blot signal was quantified using Li-COR Odyssey ImageStudio software and used to calculate the pSTAT3/STAT3 ratio. Data are shown as mean ± SEM of n = 3 biological replicates, **P < 0.01. α-tubulin was used as a loading control. (*D*) CellTiter-Glo® assay on WT and SOCS3 knockout HCC44 cells. Cells were treated with a range of concentrations of RBN2397 for 6 d. Data are shown as mean ± SEM of n = 4 biological replicates (with 2 technical replicates each).

We next investigated potential RBN2397 sensitizers. In total, we detected 305 depleted genes across the three cell lines (Fig. 3 *A* and *B* and *SI Appendix*, Figs. S2–S4). Strikingly, the only gRNAs enriched in all three cell lines corresponded to SOCS3 (suppressor of cytokine signaling 3), suggesting that it is a unique sensitivity hit in the cell lines we tested.

SOCS3 is an important regulator of immune responses and its global knockout results in embryonic lethality in mice due to placental insufficiency (35, 36). Upon binding to specific tyrosine phosphorylated cytokine receptors, such as the IL6 receptor gp130 (IL6ST), SOCS3 inhibits JAK1/2-mediated phosphorylation of downstream substrates, which include the transcription factor STAT3 (37, 38).

SOCS3 was previously detected as one of dozens of sensitivity hits in an RBN2397 CRISPR screen performed in an independent study with the H1373 cell line (Fig. 3*A*) (Dataset S4) (24). However, the authors did not report follow-up experiments to validate and characterize these hits further. SOCS3 is a direct transcriptional target of AHR, and ARNT controls SOCS3 and STAT3 signaling in an isoform-specific manner, thereby linking AHR and SOCS3 signaling with PARP7 inhibitor sensitivity (27, 39, 40). Interestingly, a zinc transporter SLC39A1 emerged as a sensitivity hit shared by SKMES1 and H838 cells (Fig. 3*B*) and shows the strongest codependency with SOCS3 according to DepMap. SOCS3 expression is controlled by zinc and intracellular zinc depletion results in reduced SOCS3 expression (41). Intrigued by these findings, we focused on the potential SOCS3-mediated PARP7i sensitivity and the interplay between SOCS3 and PARP7 signaling.

To validate the potential synthetic lethal interaction between PARP7 inhibition and SOCS3 deletion and to understand this functional link between PARP7 and SOCS3, we chose to focus on the HCC44 cell line which was the most sensitive to SOCS3 deletion and concurrent PARP7 inhibition according to our screen. SOCS3 knockout cells were generated using CRISPR/Cas9 genome editing. SOCS3 knockout cells were then analyzed together with wild-type HCC44 cells with and without PARP7 inhibitor treatment for 24 h (Fig. 3*C*). Consistent with the role of SOCS3 in the suppression of STAT3 signaling, phospho-STAT3 levels were induced in the SOCS3 knockout cell lines (Fig. 3*C*). PARP7 inhibition resulted in a robust increase in PARP7 expression in both wild-type and SOCS3 KO cell lines, validating target engagement.

Since AHR is ADP-ribosylated by PARP7 (15), we investigated whether this is the case for SOCS3. We coexpressed FLAG-tagged SOCS3 and GFP-tagged PARP7 in HEK293T cells, followed by a pulldown with anti-FLAG beads. While the positive control, androgen receptor, was ADP-ribosylated by PARP7 consistent with a previous report (21), SOCS3 was not modified (*SI Appendix*, Fig. S15). This suggests that SOCS3 is not regulated directly by PARP7-mediated ADPr.

We then investigated the effects of SOCS3 loss on PARP7 inhibitor sensitivity. The viability of SOCS3 knockout HCC44 cells measured in a luminescence assay after 6 d of RBN2397 treatment was reduced compared to wild-type HCC44 cells (Fig. 3*D*). Notably, treatment of wild-type and SOCS3 knockout HCC44 cells with a range of concentrations of a PARP1/2 inhibitor olaparib did not substantially affect cell viability (*SI Appendix*, Fig. S16). Moreover, there was no difference in viability between wild-type and SOCS3 knockout cells suggesting that PARP7-specific signaling is involved in the synthetic lethality. We validated our findings by generating a SOCS3 knockout H838 line which also had increased sensitivity to RBN2397 (*SI Appendix*, Fig. S17). Together, our results indicate that joint PARP7 inhibition and SOCS3 deletion lead to enhanced lung cancer growth suppression.

### Loss of SOCS3 Deregulates the AHR Pathway.

**Fig. 4.**
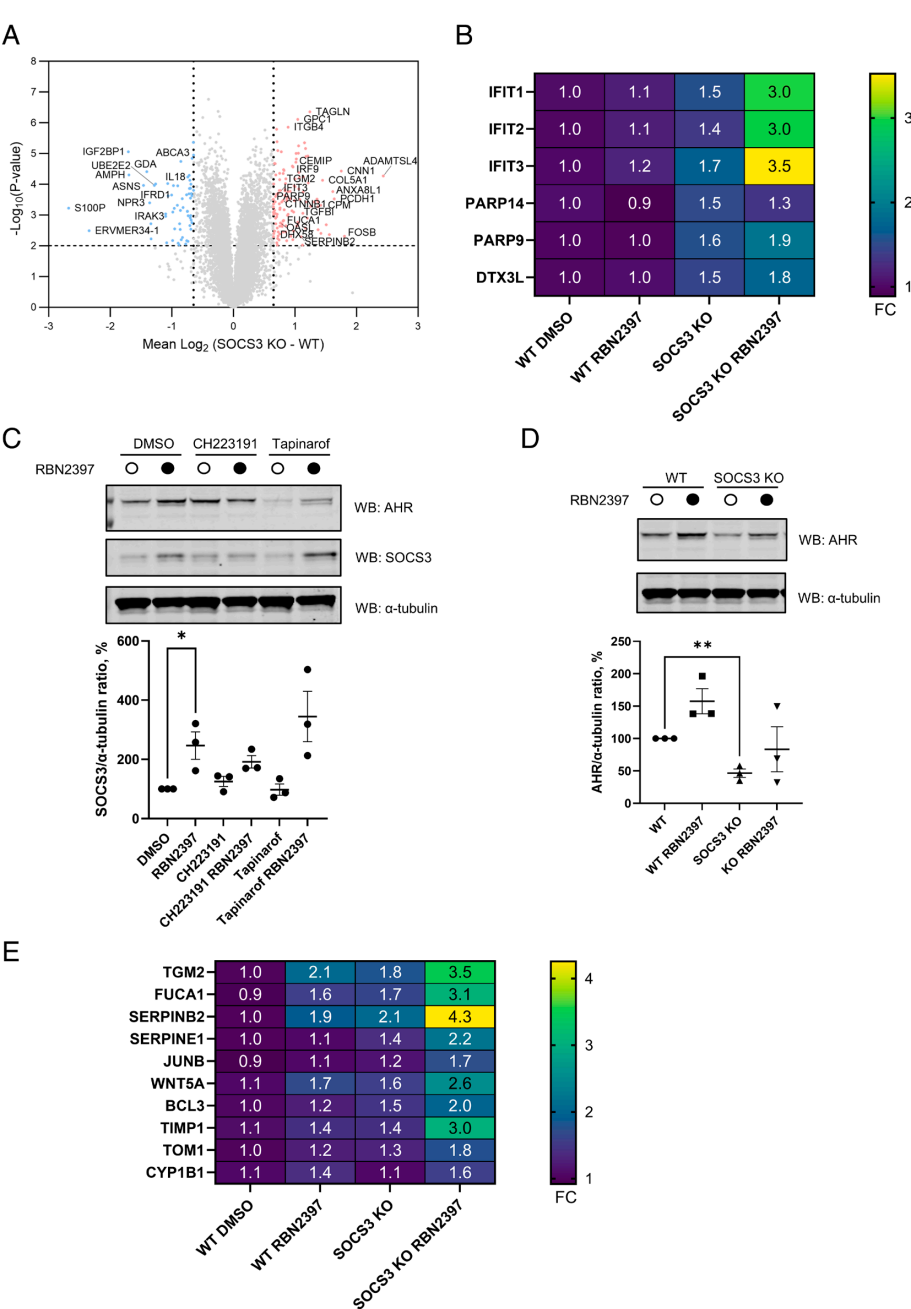
SOCS3 knockout boosts IFN- and AHR-regulated proteins upon PARP7 inhibition. (*A*) Volcano plot showing significantly upregulated (red) and downregulated (blue) proteins in SOCS3 KO compared to wild-type HCC44 cells. (*B*) Heatmap of proteomic changes to IFN-regulated proteins in SOCS3 knockouts compared to wild-type HCC44 cells with and without 1 µM RBN2397 treatment for 24 h. (*C*) Western blotting analysis of AHR and SOCS3 levels in HCC44 cells treated with 1 µM RBN2397 with or without 1 µM CH223191 and 1 µM tapinarof. Western blot signal was quantified using Li-COR Odyssey ImageStudio software. Data are shown as mean ± SEM of n = 3 biological replicates, *P < 0.05. α-tubulin was used as a loading control. (*D*) Western blotting analysis of AHR levels in wild-type and SOCS3 KO HCC44 cells treated with RBN2397 or DMSO control. Western blot signal was quantified using Li-COR Odyssey ImageStudio software. Data are shown as mean ± SEM of n = 3 biological replicates, **P < 0.01. α-tubulin was used as a loading control. (*E*) Heatmap of proteomic changes to AHR-regulated proteins (induced by the tapinarof/RBN2397 combination treatment in Fig. 2*F*) in SOCS3 knockouts compared to wild-type HCC44 cells with and without 1 µM RBN2397 treatment for 24 h.

To further characterize the SOCS3 knockout HCC44 cells we performed whole-proteome profiling using TMT-multiplexing. We observed up- and down-regulation of multiple proteins compared to wild-type control HCC44 cells (Fig. 4*A*). The levels of interferon-induced proteins IFIT3, IRF9, DHX58, OASL, PARP14, PARP9, and DTX3L were upregulated consistent with SOCS3 function in immunity (Fig. 4*A*). Interestingly, treatment of SOCS3 knockout cells with RBN2397 further boosted IFIT1, 2, and 3 levels, as well as mildly increasing PARP9 and DTX3L suggesting a synergistic mechanism (Fig. 4*B*).

We observed that HCC44 cells exhibited induction of SOCS3 levels in response to RBN2397 treatment (Fig. 4*C*). To investigate whether this was due to AHR activity, we treated wild-type cells with CH223191 and tapinarof alone or in combination with RBN2397 (Fig. 4*C*). AHR antagonist CH223191 had little effect on the SOCS3 increase induced by RBN2397 while the tapinarof/RBN2397 resulted in a small increase which was not statistically significant. These data suggest that SOCS3 expression in HCC44 cells is modulated primarily via PARP7 and not AHR.

We next wondered whether AHR itself is affected in SOCS3 knockout cells. Surprisingly, we found that AHR levels were decreased in SOCS3 knockouts (Fig. 4*D*), suggesting potential activation-induced downregulation as observed with tapinarof (Fig. 4*C*). Our proteomics data supported this as several AHR targets were further increased in SOCS3 KOs, such as SERPINB2 and SERPINE1 (Fig. 4*E*). TGM2 and FUCA1 were among the most induced proteins which was similar to that observed with combined tapinarof/RBN2397 treatment (Fig. 2 *E* and *F*).

Our data suggest a close crosstalk between SOCS3 and AHR, where PARP7 mediates SOCS3 expression and SOCS3 regulates AHR signaling.

## Discussion

PARP7 has important functions in the regulation of AHR signaling and immune responses but little is known about the pathways that are engaged in this. Unlike other IFN-induced PARPs that positively regulate IFN production and innate immunity (e.g., PARP14), PARP7 is suggested to dampen IFN responses (4, 24, 42). Here, we provide an important resource by way of CRISPR screens and proteomics to understand the molecular function of PARP7.

Others have shown that simultaneous PARP7 inhibition and AHR activation traps both of these proteins in the nucleus (23, 43). This is consistent with the extensive remodeling of the AHR-driven proteome we observed. One of the consequences of this is the strong upregulation of ASB2 E3 ligase in HCC44 cells. This ligase is thought to specifically degrade filamins A and B (34) and gratifyingly these two filamins are one of the most downregulated proteins in our proteomics experiment. Together with other proteome-wide changes, this specific pathway may be one of the key determinants of reduced cell viability observed with the tapinarof/RBN2397 treatment.

While PARP7 inhibition has been shown to restore IFN response, some cell lines like HCC44 may not respond to PARP7 inhibitors in this way. Our findings suggest that SOCS3 may dampen PARP7i-mediated IFN response in HCC44 cells. In addition, we have identified SOCS3 as a synthetic lethality hit across HCC44, SKMES1, and H838 cell lines in our screen, and this was also observed by others in another lung cancer cell line H1373 (24). These findings may have strong implications for restoring anti-tumor immunity in vivo and are supported by the report that the deletion of SOCS3 in 786-O xenografts can enhance the growth-inhibitory effect of IFN-alpha in vitro and in vivo (44). This suggests that in certain cellular contexts, as demonstrated with HCC44 cells, the depletion or inactivation of SOCS3 may further enhance the anti-tumor effect of PARP7 inhibition.

For future studies, it is important to consider that although RBN2397 had minimal effects on PARP1 activity (as demonstrated in HCC44 cells), other PARPs structurally similar to PARP7—such as PARP12 [with previously reported nanomolar IC_50_ values for RBN2397 in vitro (24)], may also be inhibited by this compound at the near-micromolar concentrations used in our CRISPR screens.

The AHR/SOCS3 pathway is an exciting link to PARP7 signaling (45, 46). We find that not only does PARP7 inhibition promote SOCS3 expression but also that SOCS3 loss deregulates AHR targets in HCC44 cells. The connection between PARP7 and SOCS3 may expand beyond AHR signaling since both have been associated with pathways controlled by other receptors such as liver X receptor, androgen receptor and estrogen receptor (16–18, 23, 39, 47–49). Moreover, PARP7 inhibition coupled with SOCS3 disruption can be explored not only as an anticancer mechanism but also as an antiviral strategy. PARP7 was suggested to be a potential target in blocking coronavirus infection and certain viruses suppress IFN responses by upregulating the host SOCS3 expression (50–52).

Taken altogether, our synthetic lethality and resistance screen combined with quantitative proteomics yielded useful information about AHR-driven PARP7 signaling. However, further work is needed to dissect the functions of specific PARP7-catalyzed ADPr substrates and specific sites that regulate AHR signaling and interferon responses. Our data will provide a valuable resource to study both AHR and PARP7 signaling for the wider community.

## Materials and Methods

### Cell Culture.

SKMES1 and NCI-H838 cell lines were purchased from the American Type Culture Collection (ATCC, Manassas, VA). HCC44 cell line was from the German Collection of Microorganisms and Cell Cultures (DSMZ, Braunschweig, Germany). All cells were cultured in a humidified 37 °C incubator at 5% (v/v) CO_2_ atmosphere. SKMES1 and HEK293T cells were cultured in DMEM (with GlutaMAX) supplemented with 10% FBS, and penicillin-streptomycin (100 U/ml and 100 μg/ml, respectively). NCI-H838 and HCC44 cells were cultured in RPMI1640 medium supplemented with 10% FBS, and penicillin-streptomycin (100 U/ml and 100 μg/ml, respectively).

### Cell Viability Assays.

CellTiter-Glo® assay (Promega G7571) was used to assess cell viability following exposure of the cells to different concentrations of RBN2397 (MedChemExpress). The experiments were carried out in white 96-well flat and clear bottom plates (Corning) with two or three technical replicates. For assays in Fig. 1 NCI-H838, SKMES1, and HCC44 cells, 1,000 cells were seeded in 50 μl of medium and grown for 24 h. The following day, 50 μl of culture medium containing twice the concentration of inhibitors or DMSO control were added to the wells. Additionally, cells were treated with a combination of Puromycin (10 μg/ml) and Staurosporine (1 μg/ml) as a positive control (maximum cell death). For HCC44 SOCS3 KO assay, 300 cells were seeded for the respective wild-type control and knockout lines. Plates were incubated at 37 °C for 6 d after which CellTiter-Glo® Substrate and CellTiter-Glo® Buffer were mixed to produce CellTiter-Glo® Reagent which was added in a 1:1 ratio to the wells (50 μl). Subsequently, the plate was incubated on a rocker for 20 min (protected from light) at room temperature. Luminescence was measured using the SpectraMax® M5 Microplate Reader (Molecular Devices LLC). GraphPad Prism (v.10) was used to plot graphs and calculate IC50s for each compound using nonlinear regression analysis (four-parameter least squares fit).

### Lentivirus Production.

Lentivirus production was performed as described in (53). Briefly, 20 M HEK293T cells were seeded in a T175 flask for ~90% confluency. The next day, cells were transfected with 30 µg transfer vector, 25 µg psPax2, and 10 µg pMD2.G, complexed with 195 µl X-tremeGENE HP in 3 ml Opti-MEM. After 20 min incubation, complexes were added to cells. The media were replaced with 30 ml DMEM the next morning. ~55 h posttransfection, lentiviral supernatant was harvested, filtered (0.45 µm), aliquoted, and stored at −80 °C.

### Genome-Wide PARP7 Inhibitor CRISPR Screens.

HCC44-Cas9 (doubling time ~42 h), SKMES1-Cas9 (doubling time ~45 h), and NCI-H838-Cas9 (doubling time ~44 h) cell lines were transduced with Yusa_v3 gRNA library in the presence of 8 µg/ml polybrene to achieve a transduction rate of ~30%. Three days post transduction, the percentage of BFP+ cells was checked by flowcytometry. Puromycin selection was started and maintained for 4 d until %BFP was >90%. At that point a baseline pellet was collected from all cell lines. Cells were then split into the dimethyl sulfoxide (DMSO) and RBN-2397 (MedChemExpress) treatment arms with two technical replicates in each. RBN-2397 was used at a concentration of 900 nM across all cell lines to achieve a growth inhibition between 10 to 25%. During the screen, DMSO treated control cells, and RBN-2397 treated cells were split every 3/4 d. Cell pellets were collected on day 11 and day 18, representing a midpoint and endpoint of the screen.

### Genomic DNA Isolation and Next Generation Sequencing (NGS).

Libraries for next generation sequencing were generated as described in (53). Briefly, gDNA was isolated (Qiagen Blood Maxi Kit) and gRNA-containing lentiviral cassettes were amplified from 5 µg gDNA using Q5 Hot Start Master Mix and primers:

Forward 5’ ACACTCTTTCCCTACACGACGCTCTTCCGATCTCTTGTGGAAAGGACGAAACA 3’, Reverse 5’ GTGACTGGAGTTCAGACGTGTGCTCTTCCGATCTACCCAGACTGCTCATCGTC 3’

PCR products were pooled, purified (Qiagen PCR Purification Kit), and used (2.5 ng) for dual-indexed Illumina library preparation (Takara HT Dual Index kit). Libraries were purified (AMPure XP beads), quantified (Qubit), and sequenced on NovaSeq6000 (PE50bp, 30% PhiX).

### CRISPR Screen Analysis.

For each CRISPR pooled screen, DNA sequencing data were generated by the Illumina HiSeq 4000 platform and FASTQ files were produced using bcl2fastq2. The reads were mapped to the Yusa v3 6-guide reference library and count data were processed through MAGeCK version 0.5.9.5 using its Test function with samples treated with RBN2397 as “test” samples and samples treated with DMSO as “controls.” For each RBN2397 versus DMSO contrast, gene-level log2 fold-changes and adjusted p-values were extracted and compared through timepoints and cell lines.

### Generation of HCC44 CRISPR/Cas9 SOCS3 Knockouts.

HCC44 SOCS3 CRISPR clones were generated as described previously (54) using Human SOCS3 CRISPR/Cas9 KO and Human SOCS3 HDR plasmids (Santa Cruz Biotechnology, #sc-400455). Transfection of Cas9-gRNA and HDR (puromycin resistance) plasmids was performed using Lipofectamine™ 3000 (Invitrogen™, #L3000008) according to the manufacturer’s instructions. 20 to 24 h later, modified cells were selected with 2 μg/ml puromycin (Invivogen) with appropriate media changes in transfected and untransfected control wells. Puromycin resistant cells were then passaged into 96-well plates at single clone density. Single colonies were identified and expanded. SOCS3 KO was validated by western blotting with a SOCS3-specific antibody.

### Western Blotting.

Cells were seeded in 6-well plates (Corning). Once 80 to 90% confluent, cells were treated with RBN2397 (1 μM final concentration) for 24 h, washed in Dulbecco’s Phosphate Buffered Saline, scraped, and centrifuged at room temperature (3,000 rpm for 4 min) to obtain pellets. Pellets were lysed immediately by resuspension in 50 μl lysis buffer (50 mM Tris pH 8, 100 mM NaCl, 1% Triton X-100 pH 8, protease and phosphatase inhibitor, 1 μM olaparib, 1 μM PARG inhibitor (PDD00017273, Sigma Aldrich), 2 μM benzonase) and incubated on ice for 25 min to extract proteins. Lysates were subsequently centrifuged at 4 °C (13,500 rpm for 10 min) to supernatants. Protein concentration was determined using the Pierce™ BCA Protein Assay Kit (ThermoFisher Scientific, #23225) according to the manufacturer’s instructions and adjusted to 1 μg/μl by adding the required volume of MilliQ H_2_O and NuPAGE® 1X LDS Sample Buffer with Bond-BreakerTM TCEP solution (both from ThermoFisher Scientific). Samples were then boiled at 95 °C for 5 min. Samples were loaded on a NuPAGETM 4-12% Bis-Tris Gels (Invitrogen). Gels were subsequently subjected to SDS-PAGE at 160 V in MOPS buffer before transfer to a Trans-Blot Turbo Transfer membrane (Bio-Rad) using a Trans-Blot Turbo Transfer System (Bio-Rad) (standard 30 min transfer). Ponceau S staining (Sigma) was used to determine transfer efficiency. Membranes were blocked using 3% (w/v) bovine serum albumin (BSA) in 1X phosphate-buffered saline-0.1% (w/v) Tween® 20 (PBS-T) for 1 h. Membranes were subsequently incubated in primary antibodies overnight at 4 °C, followed by secondary antibody incubation for 1 h at room temperature. Antibody list: SOCS3 (Proteintech, 14025-1-AP, 1:1,000, rabbit), phospho-STAT3 (Cell Signaling Technology #9145, 1:1,000, rabbit), STAT3 (Cell Signaling Technology #9139, 1:1,000, mouse), AHR (Santa Cruz, sc-133088, 1:1,000, mouse), alpha-tubulin (Proteintech, 11224-1-AP, 1:2,000, rabbit), STAT1 (Cell Signaling Technology #9172, 1:1,000, rabbit), phospho-STAT1 (Cell Signaling Technology #9167, 1:1,000, rabbit), ANLN (Santa Cruz, sc-271814, 1:400, mouse), SERPINB2 (Proteintech 16035-1-AP, 1:1,000, rabbit), SERPINE1 (PAI1, Proteintech 13801-1-AP, 1:1,000, Rabbit), CYP1B1 (Proteintech, 67033-1-Ig, 1:1,000, mouse), filamin A (Cell Signaling Technology, #44873, 1:1,000, rabbit), filamin B (Proteintech, 20685-1-AP, 1:1,000, rabbit), p21/CDKN1A (Cell Signaling Technology, #2947, 1:1,000, rabbit), FOSL1/FRA1 (Cell Signaling Technology, #5281, 1:1,000, rabbit), GFP (Santa Cruz, sc-9996, 1:1,000, mouse), poly/mono-ADP-ribose (Cell Signaling Technology, #83732 (E6F6A), 1:1,000, rabbit) PARP7 (Jason Matthews laboratory, 1:1,000, mouse), goat anti-rabbit IgG secondary (800CW, 926-32211, Li-COR Biosciences), goat anti-mouse IgG secondary (680RD, 926-68070, Li-COR Biosciences). All antibodies were diluted in 3% (w/v) BSA in 1X PBS-T. Blots were visualized using the Odyssey CLx Imager (LI-COR Biosciences). Quantification was performed using the Odyssey Image Studio Lite® software v.5.2 (LI-COR Biosciences).

### Whole Proteome and Phosphoproteome Profiling.

#### Cell lysis and protein digestion.

Cells were seeded in 10 cm dishes (Corning). Once 80 to 90% confluent, cells were treated with RBN2397 (MedChemExpress), tapinarof (MedChemExpress), or CH223191 (CaymanChemical) (1 μM final concentration each) for 24 h Cells were washed with PBS twice and scraped in a lysis buffer containing 8 M urea, 200 mM EPPS, pH 8.5 with protease and phosphatase inhibitors on ice. Cells were homogenized by 12 passes through a 21-gauge (1.25 inches long) needle. The homogenate was sedimented by centrifugation at 21,000×*g* for 5 min and the supernatant was transferred to a new tube. Protein concentrations were determined using the bicinchoninic acid (BCA) assay (ThermoFisher Scientific). Proteins were subjected to disulfide bond reduction with 5 mM tris (2-carboxyethyl) phosphine (room temperature, 15 min) and alkylation with 10 mM iodoacetamide (room temperature, 20 min in the dark). Excess iodoacetamide was quenched with 10 mM dithiothreitol (room temperature, 15 min in the dark). Methanol-chloroform precipitation was performed prior to protease digestion (55). Samples were reconstituted in 200 mM EPPS buffer at pH 8.5 and digested at room temperature for 14 h using LysC protease at a protein-to-protease ratio of 100:1. Subsequently, trypsin was added at the same 100:1 protein-to-protease ratio, and the mixture was incubated at 37 °C for 6 h.

#### Tandem mass tag labeling.

TMTpro reagents (0.8 mg) were dissolved in 40 μL of anhydrous acetonitrile, and 5 μL of this solution was combined with 50 µg of peptides along with 15 μL of acetonitrile, resulting in a final acetonitrile concentration of approximately 30% (v/v). The mixture was incubated at room temperature for 1 h, after which the reaction was stopped by adding hydroxylamine to a final concentration of 0.3% (v/v). TMT-labeled samples were then combined in a 1:1 ratio across all samples. Each pooled sample was concentrated by vacuum centrifugation to near dryness and then processed using C18 solid-phase extraction (SPE) with Sep-Pak columns (Waters).

#### Spin column-based phosphopeptide enrichment.

Phosphopeptides were isolated from the pooled sample using the High-Select Fe-NTA Phosphopeptide Enrichment Kit (55). The only modification to the manufacturer’s protocol was the preparation of an “elution collection tube” containing 100 µL of 10% formic acid, into which the eluates were collected. The combined eluate was concentrated to near dryness by vacuum centrifugation and then desalted using StageTips. The unbound fraction and wash were utilized for offline basic pH reversed-phase (BPRP) fractionation to analyze the proteome, as described below.

#### Off-line basic pH reversed-phase (BPRP) fractionation (whole proteome).

The pooled TMT-labeled peptides, specifically the unbound and wash fractions from the phosphopeptide enrichment, were separated using basic pH reversed-phase (BPRP) HPLC with an Agilent 1260 pump. The peptides were eluted over a 50-min linear gradient, increasing from 5 to 35% acetonitrile in 10 mM ammonium bicarbonate at pH 8, with a flow rate of 0.8 mL/min, using an Agilent 300Extend C18 column (3.5 μm particles, 2.1 mm internal diameter, 25 cm length). The peptide mixture was divided into 96 fractions, which were then combined into 24 superfractions. From these, 12 nonadjacent fractions were selected for mass spectrometry analysis. Each of these fractions was acidified with 1% formic acid and concentrated to near dryness by vacuum centrifugation. Additionally, each fraction was desalted using StageTips, further dried by vacuum centrifugation, and resuspended in a solution of 5% acetonitrile and 5% formic acid for LC–MS/MS analysis.

#### Liquid chromatography and mass spectrometry data acquisition (whole proteome of HCC44, H838, and SKMES1 cells).

Mass spectrometry data for 24 (HCC44) or 12 nonadjacent (H838, SKMES1) superfractions were collected using an Orbitrap Exploris 480 mass spectrometer (Thermo Fisher Scientific, San Jose, CA) coupled with a nLC-1200 liquid chromatographer. Peptide separation was performed using a microcapillary column with an inner diameter of 100 μm, filled with approximately 35 cm of Accucore C18 resin (2.6 μm, 150 Å, Thermo Fisher Scientific). For each experiment, approximately 2 μg of sample was loaded onto the column. The peptides were eluted over a 90-min gradient, ranging from 5 to 29% acetonitrile in 0.125% formic acid, at a flow rate of 450 nL/min. The acquisition process started with an Orbitrap MS1 scan, set with the following specifications: resolution of 60,000, mass range of 350 to 1350 Th, automatic gain control (AGC) target at 100%, maximum injection time of 50 ms, and data recorded in centroid mode. For MS2 analysis, a cycle time of 1 s was applied, utilizing HCD (high-energy collision dissociation) with the following settings: resolution of 50,000, AGC of 200%, maximum injection time of 120 ms, isolation window of 0.6 Th, normalized collision energy (NCE) of 35%, and centroid spectrum data type. Dynamic exclusion was configured to operate in automatic mode. The FAIMS compensation voltages (CV) were set at −40 V, −60 V, and −80 V. A 1 s TopSpeed cycle was used for each CV.

#### Liquid chromatography and tandem mass spectrometry (phosphoproteome in HCC44 cells).

Data acquisition was performed using an Orbitrap Eclipse mass spectrometer connected to a Vanquish Neo UHPLC system. About 1 µg of peptide was fractionated at a flow rate of 300 nL/min on a 100 µm capillary column, which was packed with 35 cm of Accucore 150 resin (2.6 μm, 150 Å; Thermo Fisher Scientific). The gradient was 7 to 25% Buffer B (95% acetonitrile, 0.125% formic acid) which was mixed into buffer A (5% acetonitrile, 0.125% formic acid) over a 150 min gradient. The acquisition sequence started with an Orbitrap MS1 scan, configured with the following settings: resolution of 60,000, mass range of 350-1350 Th, automatic gain control (AGC) set to 100%, and maximum injection time of 118 ms. For MS2 analysis, higher-energy collisional dissociation (HCD) was used with the following specifications: resolution of 50,000, AGC of 300%, normalized collision energy (NCE) of 36%, maximum injection time of 250 ms, and isolation window of 0.7 Th. Additionally, unassigned, singly charged, and species with charges greater than 5 + were excluded from MS2 analysis, and dynamic exclusion was set to 90 s. Data were acquired using two injections, one with a CV set of −40/−60/−80 V and a second injection with a CV set of −30/−40/−50 V. A 1 s TopSpeed cycle was used for each CV.

#### Liquid chromatography and tandem mass spectrometry (phosphoproteome in H838 and SKMES1 cells).

Mass spectrometry data were acquired using an Orbitrap Ascend MultiOmics mass spectrometer linked to a Vanquish Neo UHPLC system. Roughly 1 µg of peptide was fractionated at a flow rate of 300 nL/min on a 100 µm capillary column, filled with 35 cm of Accucore 150 resin (2.6 μm, 150 Å; Thermo Fisher Scientific). The gradient was 7 to 25% Buffer B (95% acetonitrile, 0.125% formic acid) which was mixed into buffer A (5% acetonitrile, 0.125% formic acid) over a 135 min gradient. The scan sequence began with an MS1 spectrum (Orbitrap analysis, resolution 60,000, 350 to 1350 Th, automatic gain control (AGC) target is set to 100%, maximum injection time set to 50 ms). The hrMS2 stage consisted of fragmentation by higher energy collisional dissociation (HCD, normalized collision energy 36%) and analysis using the Orbitrap (AGC 200%, maximum injection time 120 ms, isolation window 0.7 Th, resolution 45,000 with TurboTMT activated). Data were acquired using the FAIMSpro interface, the dispersion voltage (DV) set to 5,000 V, the compensation voltages (CVs) were set at −30 V, −50 V, and −70 V. The sample was analyzed twice with the second acquisition using a different set of CV (−40 V, −60 V, and −80 V). The TopSpeed parameter was set at 1 s per CV.

#### Liquid chromatography and tandem mass spectrometry (wild-type and SOCS3 knockout HCC44 cells).

Mass spectrometry data were collected using a Orbitrap Astral mass spectrometer (Thermo Fisher Scientific, San Jose, CA) coupled with Neo Vanquish liquid chromatograph. Peptides were separated on a 110 cm µPAC C18 column (Thermo Fisher Scientific). For each analysis, we loaded ~0.5 μg onto the column. Peptides were separated using a 90 min gradient of 5 to 29% acetonitrile in 0.125% formic acid with a flow rate of 250 nL/min. The acquisition sequence started with an Orbitrap MS1 scan, configured with the following settings: resolution of 60,000, mass range of 350 to 1350 Th, automatic gain control (AGC) set to 100%, and maximum injection time of 118 ms. For MS2 analysis, higher-energy collisional dissociation (HCD) was used with the following specifications: resolution of 50,000, AGC of 300%, normalized collision energy (NCE) of 36%, maximum injection time of 250 ms, and isolation window of 0.7 Th. Additionally, unassigned, singly charged, and species with charges greater than 5+ were excluded from MS2 analysis, and dynamic exclusion was set to 90 s.

#### Data analysis.

Spectra were converted to mzXML via Msconvert (56). Database searching included all entries from the human UniProt reference Database (downloaded: April 2023). The database was concatenated with one composed of all protein sequences for that database in reversed order. Searches were performed using a 50-ppm precursor ion tolerance for total protein level profiling and the product ion tolerance was set to 0.03 Da. These wide mass tolerance windows were chosen to maximize sensitivity in conjunction with Comet searches and linear discriminant analysis (57, 58). TMTpro labels on lysine residues and peptide N termini +304.207 Da), as well as carbamidomethylation of cysteine residues (+57.021 Da) were set as static modifications, while oxidation of methionine residues (+15.995 Da) was set as a variable modification. In addition, deamidation (+0.984 Da) at glutamine and asparagine residues and phosphorylation (+79.966 Da) at serine, threonine, and tyrosine residues were also set as variable modifications for phosphopeptide enrichment. Peptide–spectrum matches (PSMs) were adjusted to a 1% false discovery rate (FDR) (59, 60). PSM filtering was performed using a linear discriminant analysis, as described previously (57) and then assembled further to a final protein-level FDR of 1% (60). For phosphosite identification, the Ascore (58) false-discovery metric was used and only phosphosites that were “high-confidence”, with *P* ≤ 0.05, were retained. Proteins and phosphorylation sites were quantified by summing reporter ion counts across all matching PSMs, also as described previously (61). Reporter ion intensities were adjusted to correct for the isotopic impurities of the different TMTpro reagents according to manufacturer specifications. The signal-to-noise measurements of peptides assigned to each protein were summed and these values were normalized so that the sum of the signal for all proteins in each channel was equivalent. Each protein abundance measurement was scaled, such that the summed signal-to-noise for that protein across all channels equals 100.

### Transfection and Pulldown using GFP and FLAG beads.

HEK293T cells were seeded at 0.8 * 106 cells per well in a 6-well plate. The following day cells were transfected with 1 µg of GFP-PARP7, GFP-AR, or SOCS3-FLAG plasmids and 5 ul of PolyFect (QIAGEN) diluted in 200 ul of OPTI-MEM. 24 h posttransfection cells were treated with DMSO or 1 µM RBN2397 for a further 24 h. Cells were lysed as described in the western blotting section. Lysates were incubated with anti-GFP beads (Chromotek) or anti-FLAG M2 beads (Sigma Aldrich) for 2 h at 4 °C. The beads were washed five times with TBS containing 0.5 % Triton X-100 and boiled in 2xLDS supplemented with 50 mM DTT followed by western blotting.

## Supplementary Material

Appendix 01 (PDF)

Dataset S01 (XLSX)

Dataset S02 (CSV)

Dataset S03 (CSV)

Dataset S04 (XLSX)

Dataset S05 (XLSX)

Dataset S06 (XLSX)

## Data Availability

The mass spectrometry proteomics data have been deposited to the ProteomeXchange Consortium via the PRIDE (62) partner repository with the dataset identifier PXD062520 (63).
