# Supplementary material for: CRISPR screens and quantitative proteomics reveal remodeling of the aryl hydrocarbon receptor–driven proteome through PARP7 activity
Source: Proc Natl Acad Sci U S A. 2025 Jun 10;122(24):e2424985122. doi: 10.1073/pnas.2424985122 (PMC12184497; doi:10.1073/pnas.2424985122)
Supplement: Supplementary file 1 — Appendix 01 (PDF) [file pnas.2424985122.sapp.pdf]

PNAS

Supporting Information for

CRISPR screens and quantitative proteomics reveal remodeling of the aryl hydrocarbon receptor-driven proteome through PARP7 activity

Andrii Gorelik[1,2]*, Joao A. Paulo[2], Christina B. Schroeter[2,3], Melanie Lad[4], Abigail Shurr[4], Chara Mastrokalou[4], Samrah Siddiqi[1], Osamu Suyari[1], John Brognard[5], David Walter[4], Jason Matthews[6,7], Timothy M. Palmer[8], Steven P. Gygi[2] and Ivan Ahel[1]*

1 Sir William Dunn School of Pathology, University of Oxford, Oxford, OX1 3RE, UK

2 Department of Cell Biology, Harvard Medical School, Boston, MA 02115, USA

3 Department of Neurology, Medical Faculty and University Hospital Düsseldorf, Heinrich Heine University Düsseldorf, Germany

4 Cancer Research Horizons, Joint AstraZeneca-Cancer Research Horizons Functional Genomics Centre, Cambridge, UK

5 Laboratory of Cell and Developmental Signaling, Center for Cancer Research, National Cancer Institute, Frederick, MD, USA

6 Department of Nutrition, Institute of Basic Medical Sciences, University of Oslo, 0317 Oslo, Norway

7 Department of Pharmacology and Toxicology, University of Toronto, Toronto, ON M5S 1A8, Canada

8 Biomedical Institute for Multimorbidity, Centre for Biomedicine, Hull York Medical School, University of Hull, Hull HU6 7RX, UK

*Andrii Gorelik and Ivan Ahel.

Email: andrii.gorelik@path.ox.ac.uk and ivan.ahel@path.ox.ac.uk

This PDF file includes:

Figures S1 to S25
SI References

Other supporting materials for this manuscript include the following:

Datasets S1 to S6

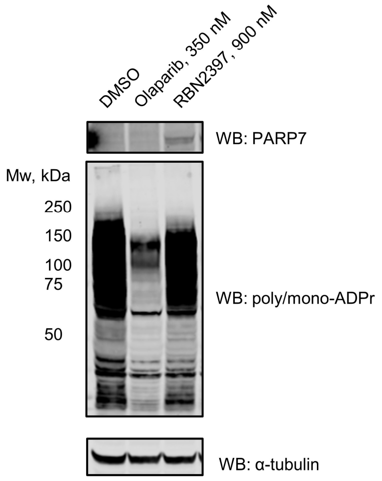

Fig. S1. Western blot of total ADP-ribosylation levels upon olaparib and RBN2397 treatments. HCC44 cells were treated with DMSO, olaparib or RBN2397 at indicated concentrations. Total ADP-ribosylation was detected with a poly-mono-ADP-ribose antibody. RBN2397 target engagement (increased protein levels) was validated by western blotting using a PARP7-specific antibody..

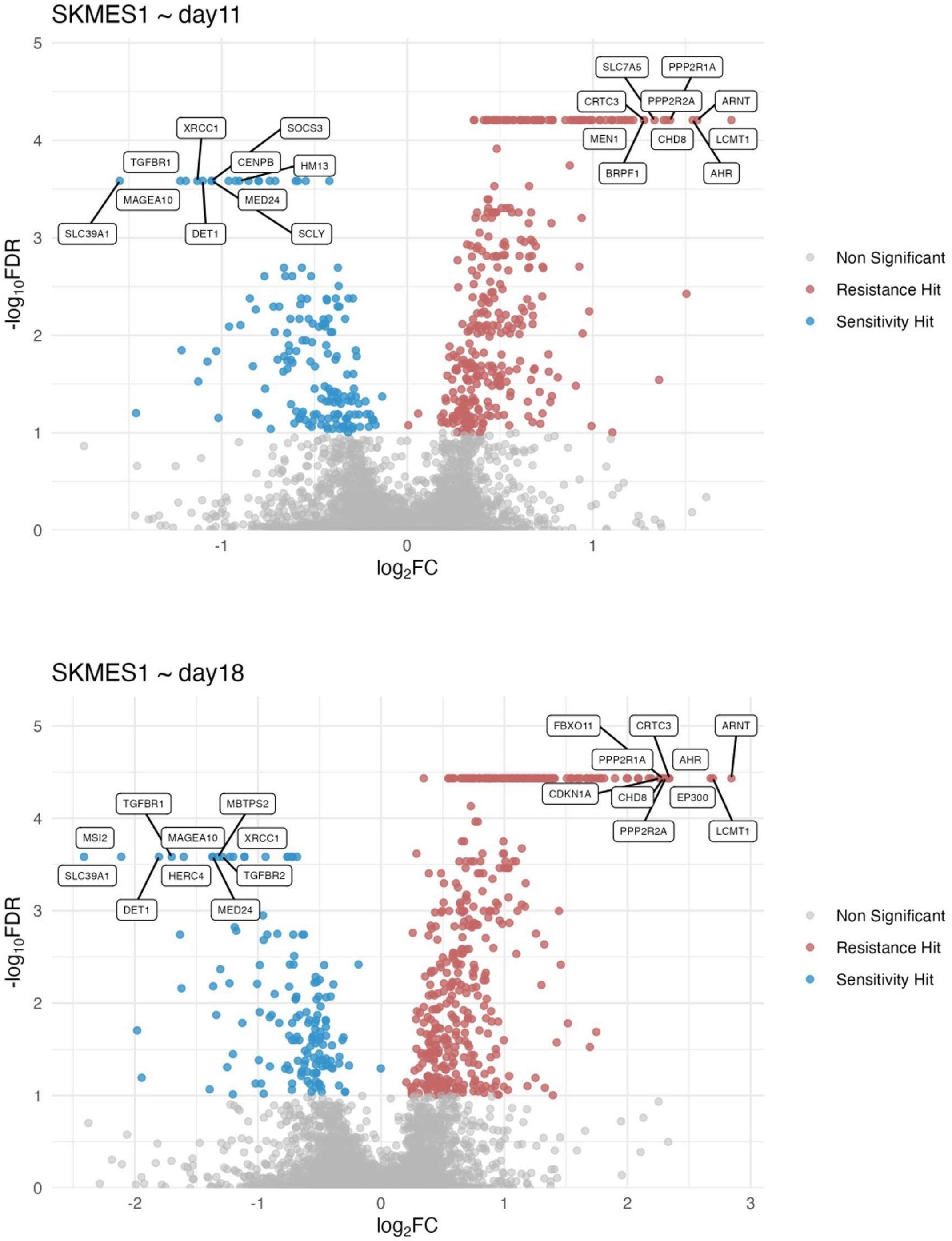

Fig. S2. Sensitivity and resistance hits in SKMES1 cells at day 11 and day 18 of the screen. Volcano plots showing significantly upregulated enriched (red, resistance) and downregulated depleted (blue, sensitivity) genes in SKMES1 cells treated with RBN2397 compared to DMSO. Genes with False Discovery Rate (FDR) < 0.1 are considered significant. The names of the top 10 most significantly enriched and depleted genes are shown.

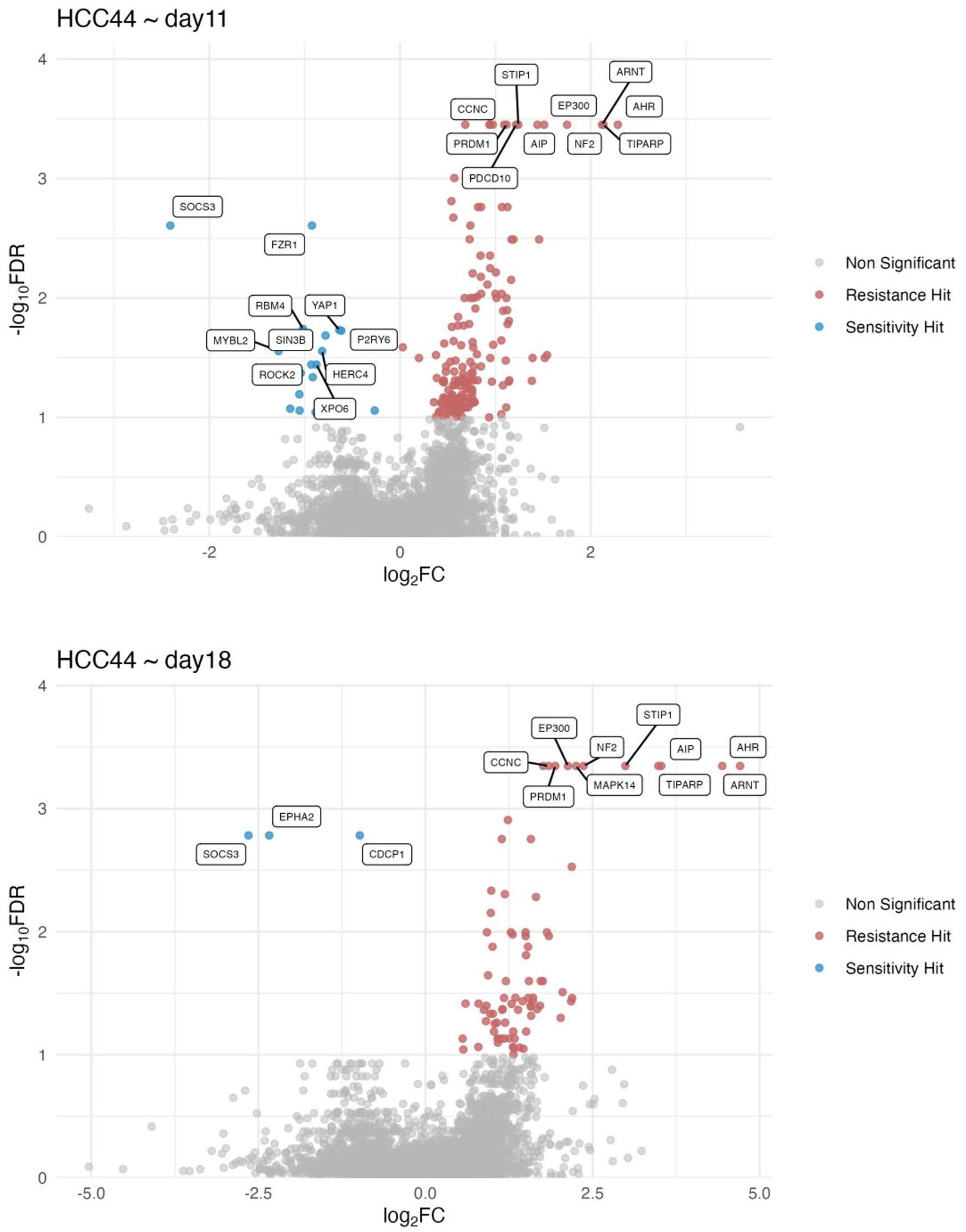

Fig. S3. Sensitivity and resistance hits in HCC44 cells at day 11 and day 18 of the screen. Volcano plots showing significantly upregulated enriched (red, resistance) and downregulated depleted (blue, sensitivity) genes in HCC44 cells treated with RBN2397 compared to DMSO. Genes with False Discovery Rate (FDR) < 0.1 are considered significant. The names of the top 10 most significantly enriched and depleted genes are shown.

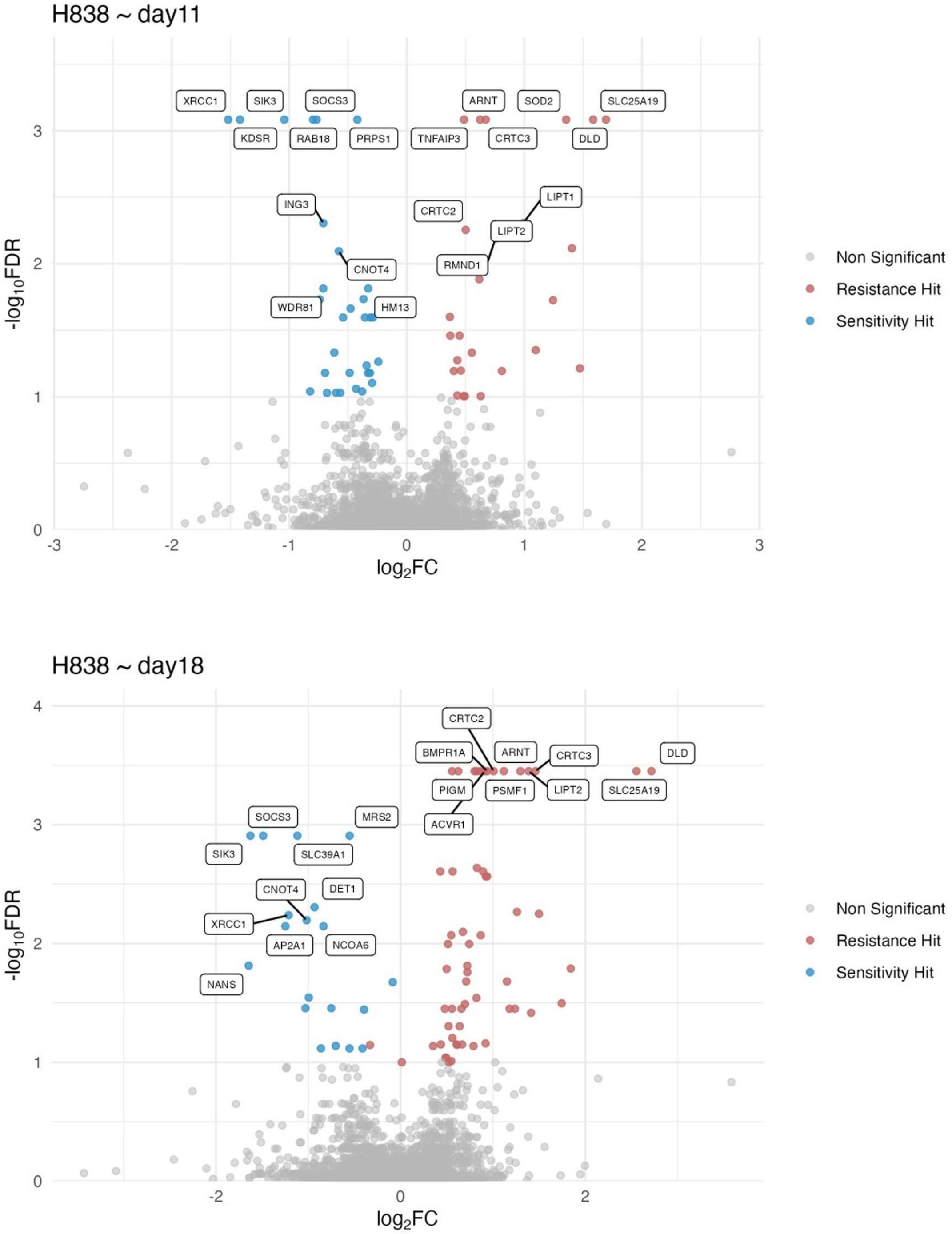

Fig. S4. Sensitivity and resistance hits in NCI-H838 cells at day 11 and day 18 of the screen. Volcano plots showing significantly upregulated enriched (red, resistance) and downregulated depleted (blue, sensitivity) genes in H838 cells treated with RBN2397 compared to DMSO. Genes with False Discovery Rate (FDR) < 0.1 are considered significant. The names of the top 10 most significantly enriched and depleted genes are shown.

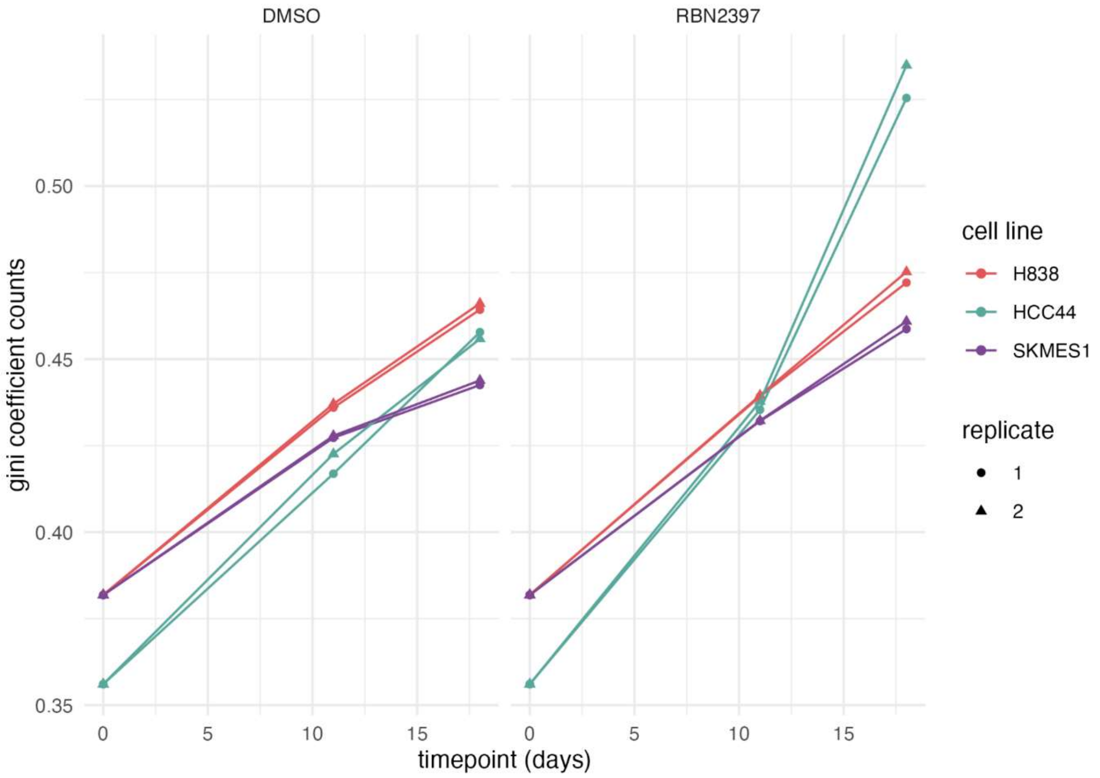

Fig. S5. Gini coefficient curves over time in the CRISPR screen. Gini coefficients reflect the effect of selection on the population of gRNAs. In positive selection experiments, Gini coefficients are expected to increase through time and due to a treatment, since a few sgRNAs with high counts can dominate the final pool while most of the other cells die. The Gini coefficient is plotted for the three cell lines (H838, HCC44, and SKMES1) over two timepoints (day 11 and 18). The plasmid library is depicted as day 0. Data are presented for both DMSO-control (left) and RBN2397-treated (right) conditions. Over time and under RBN2397 treatment, an increase in Gini coefficients is observed across all cell lines, with a more pronounced effect in HCC44.

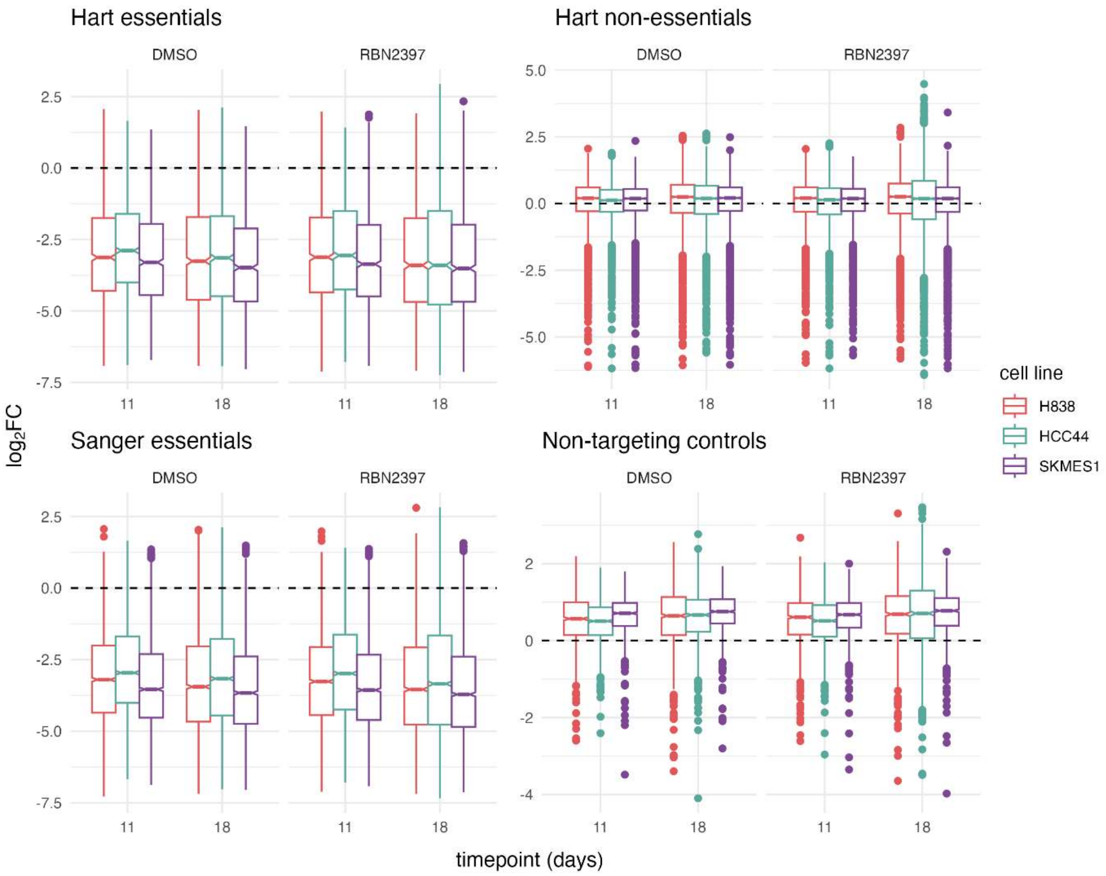

Fig. S6. Log_2FC distributions of sgRNAs in the CRISPR screen. Each panel presents changes in log_2FC of sgRNAs in indicated samples compared to the plasmid pool in response to RBN2397 treatment across three different cell lines: H838 (red), HCC44 (green), and SKMES1 (purple), at two timepoints: 11 and 18 days. Data are shown for four gene sets: Hart (1) essentials (top-left), Sanger (2) essentials (bottom-left), Hart (1) non-essentials (top-right), and Non-targeting controls (bottom-right). The essential genes (Hart and Sanger essentials) exhibit depletion, while non-essential genes and non-targeting controls remain roughly centred around zero, suggesting the effectiveness of the perturbations.

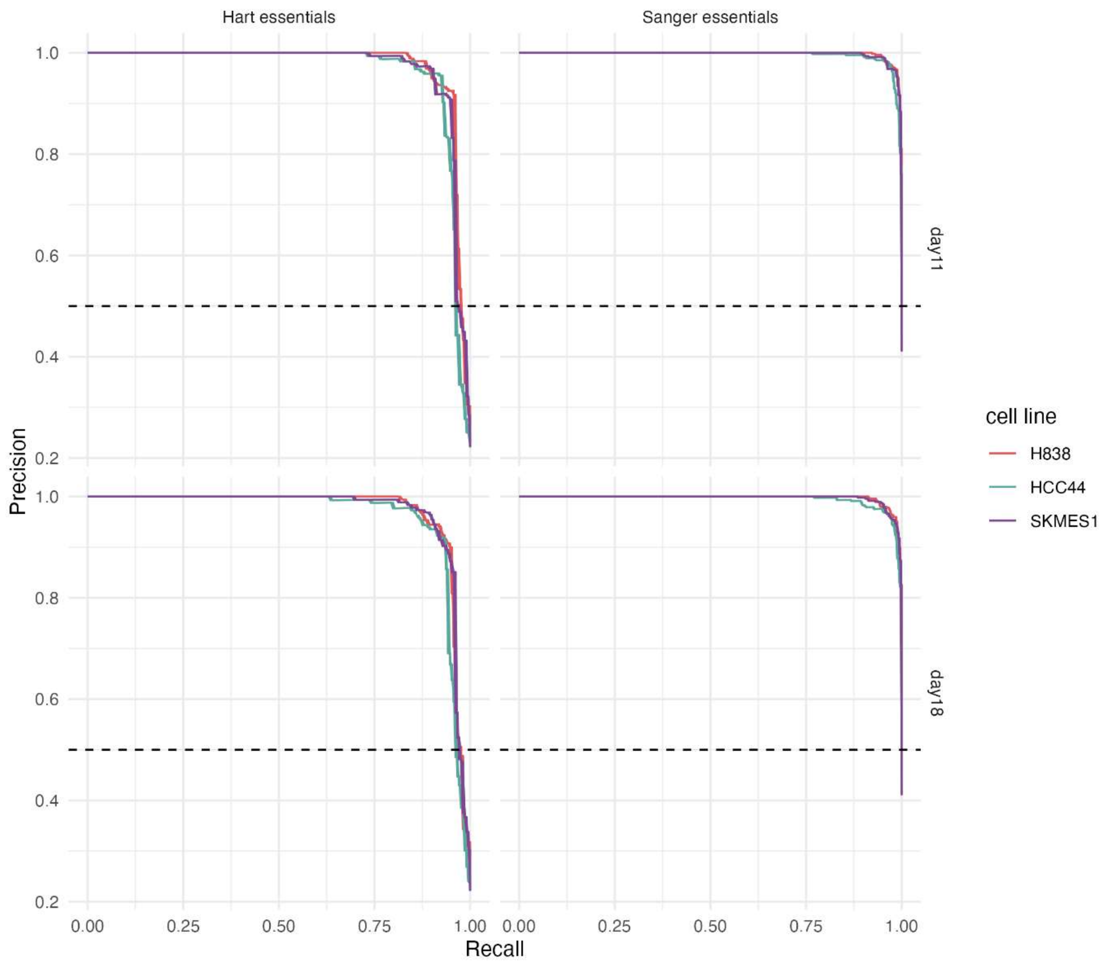

Fig. S7. Precision-recall (PR) curves assessing CRISPR screen quality. The curves were calculated using the control-DMSO samples for the three different cell lines (H838, HCC44, and SKMES1) at both timepoints (11 and 18 days). Precision is plotted against Recall to evaluate the performance of gene essentiality classification, when considering the gene BF (Bayes Factor (3)) as a rank-based classifier of reference gene sets of essential (Hart (1) and Sanger (2)) and non-essential genes (Hart (1)). The curves indicate high classification performance across all cell lines and timepoints, with minimal variation between conditions (AUPRC > 0.96 for Hart and > 0.99 for Sanger essentials).

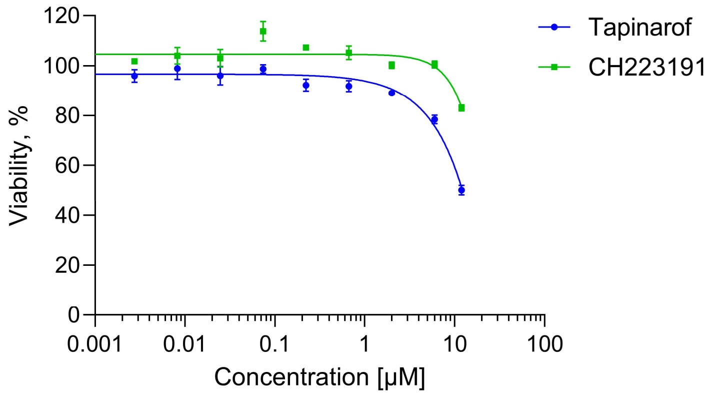

Fig. S8. Effects of tapinarof and CH223191 treatment on cell viability. HCC44 cells were treated at varying tapinarof and CH223191 concentrations. Cell viability was assessed with CellTiter-Glo®. Data are shown as mean ± s.e.m. of n = 3 replicates.

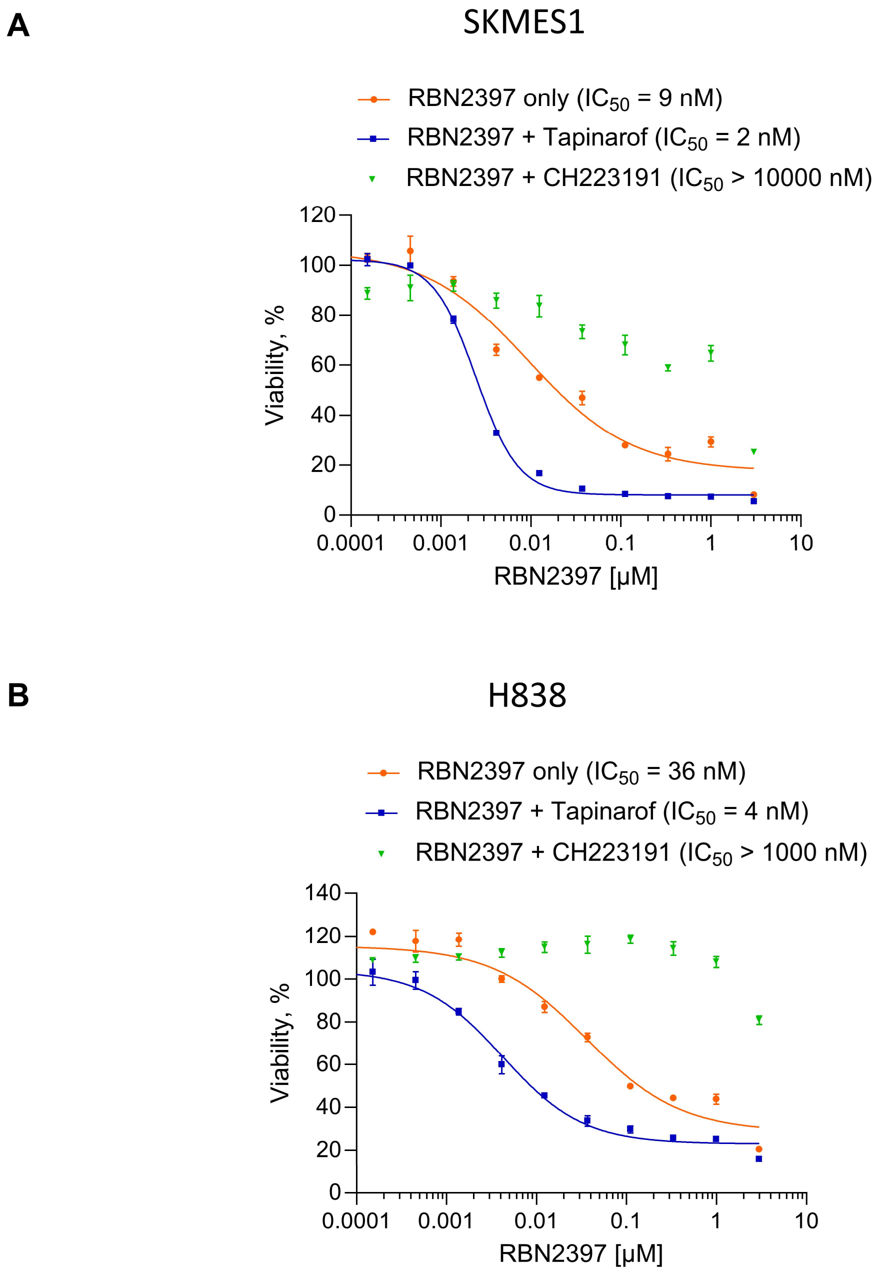

Fig. S9. Effects of tapinarof and CH223191 combination treatment with RBN2397 on cell viability. Tapinarof and CH223191 were both used at 1 µM. Cell viability was assessed with CellTiter-Glo®. Data are shown as mean ± s.e.m. of n = 3 replicates. A. Cell viability of SKMES1 cells. B. Cell viability of H838 cells.

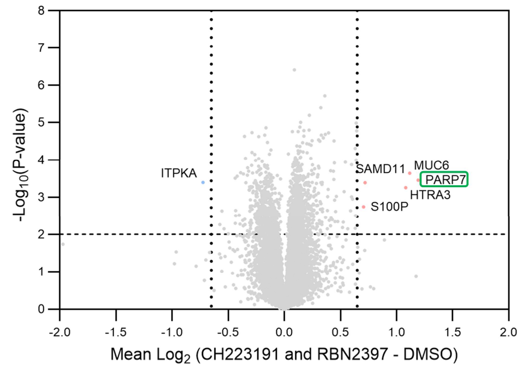

Fig. S10. Proteomic changes upon CH223191 and RBN2397 combination treatment. TMT 18-plex quantitative proteomics volcano plot showing significantly upregulated (red) and downregulated (blue) proteins in HCC44 cells treated with a combination of 1 µM RBN2397 and 1 µM CH223191 for 24 h. DMSO was used as a control.

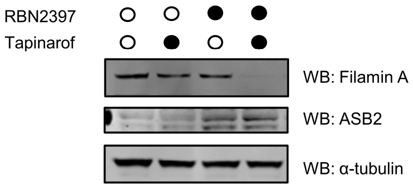

Fig. S11. Western blot validation of Filamin A degradation in HCC44 cells upon combined tapinarof and RBN2397 treatment. HCC44 cells were treated for 72 h with 1 μM tapinarof, 1 μM RBN2397 or their combination. Filamin A degradation and ASB2 upregulation were visualised by western blot. α-tubulin was used as a loading control.

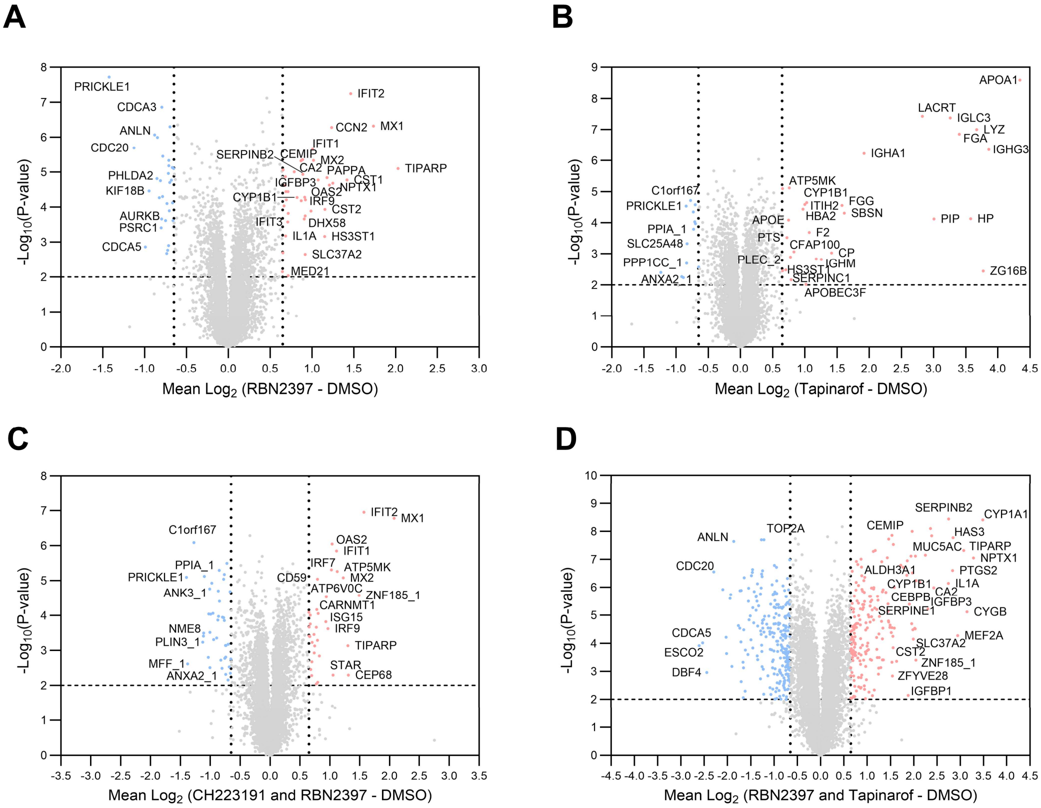

Fig. S12. Proteomic changes via AHR modulation in SKMES1 cells. TMT 18-plex quantitative proteomics volcano plots showing significantly upregulated (red) and downregulated (blue) proteins in SKMES1 cells treated with 1 µM RBN2397 (**A**), 1 µM tapinarof (**B**), and a combination of 1 µM RBN2397 and 1 µM CH223191 (**C**) or 1 µM RBN2397 and 1 µM tapinarof (**D**), respectively, for 24 h.

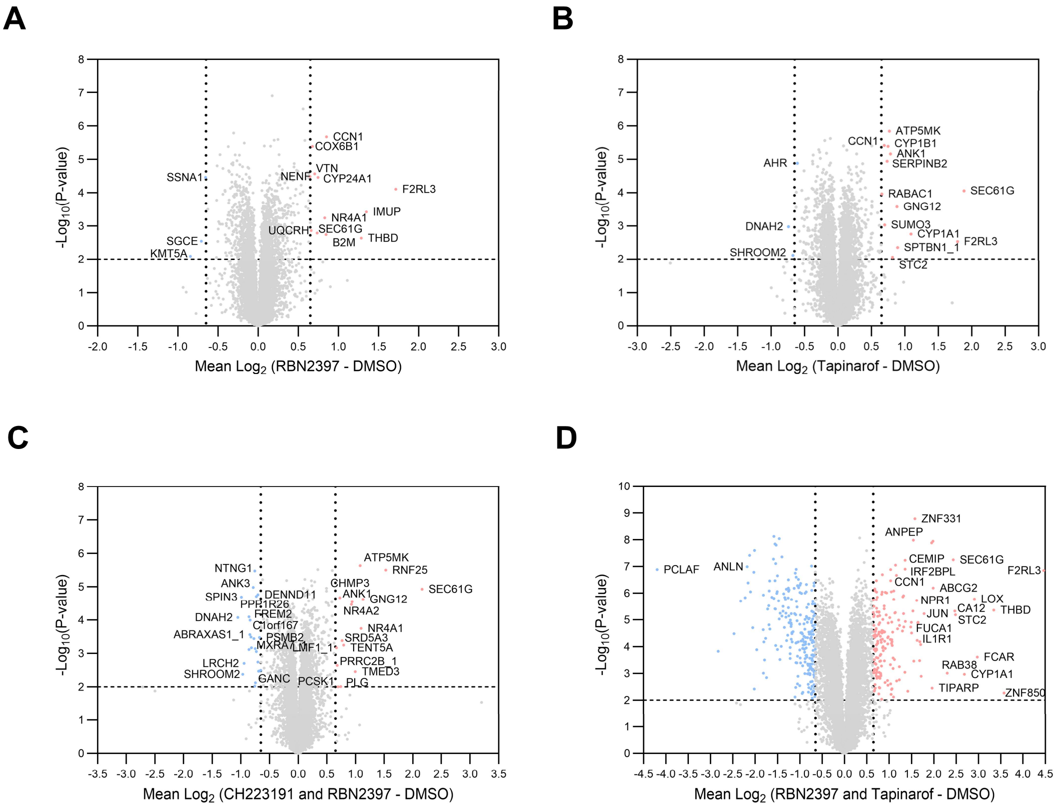

Fig. S13. Proteomic changes via AHR modulation in H838 cells. TMT 18-plex quantitative proteomics volcano plots showing significantly upregulated (red) and downregulated (blue) proteins in H838 cells treated with 1 μM RBN2397 (**A**), 1 μM tapinarof (**B**), and a combination of 1 μM RBN2397 and 1 μM CH223191 (**C**) or 1 μM RBN2397 and 1 μM tapinarof (**D**), respectively, for 24 h.

A *SKMES1*

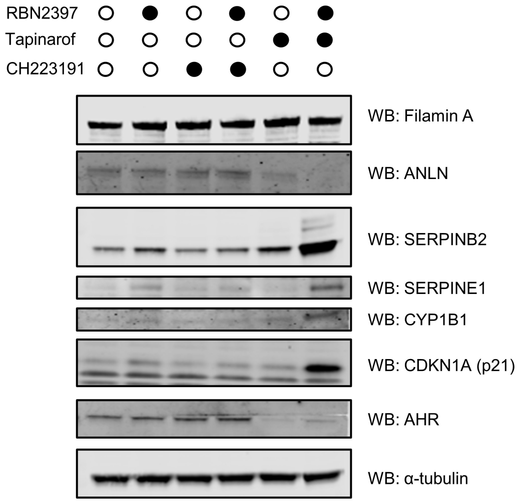

B *H838*

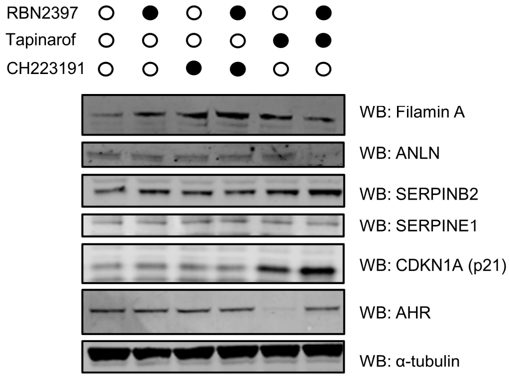

Fig. S14. Western blot validation of proteomics hits using RBN2397, tapinarof, CH223191 and combination treatments in HCC44, SKMES1 and H838 cells. A. Western blot validation of proteomics hits (Supplementary Fig. 12) in SKMES1 cells. **B**. Western blot validation of proteomics hits (Supplementary Fig. 13) in H838 cells. Cells were treated with 1 μM RBN2397, 1 μM tapinarof, 1 μM CH223191 or a combination of 1 μM RBN2397 and 1 μM CH223191, or 1 μM RBN2397 and 1 μM tapinarof for 24 h. α-tubulin was used as a loading control.

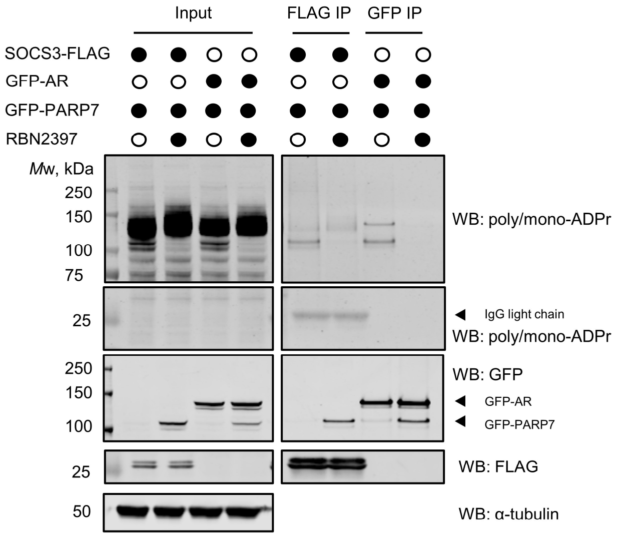

Fig. S15. SOCS3 is not ADP-ribosylated by PARP7. FLAG-tagged SOCS3 was pulled down using FLAG M2 beads. GFP-tagged androgen receptor (GFP-AR) was pulled down using GFP beads. GFP-AR was used as a positive control for PARP7-mediated ADP-ribosylation. RBN2397 treatment was performed at 1 μM for 24 h.

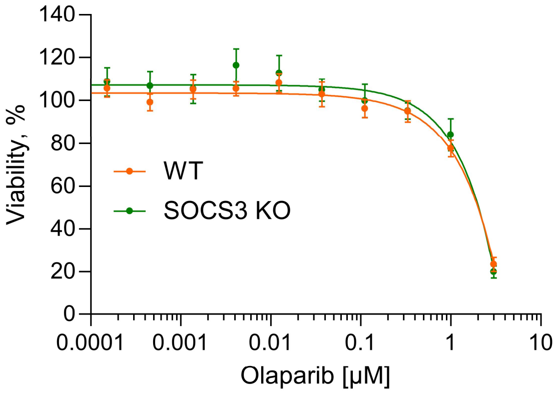

Fig. S16. Effects of olaparib treatment on the viability of wild-type and SOCS3 knockout cells. Wild-type or SOCS3 knockout HCC44 cells were treated with varying concentrations of olaparib for 6 days. Cell viability was assessed with CellTiter-Glo®. Data are shown as mean ± s.e.m. of n = 3 biological replicates.

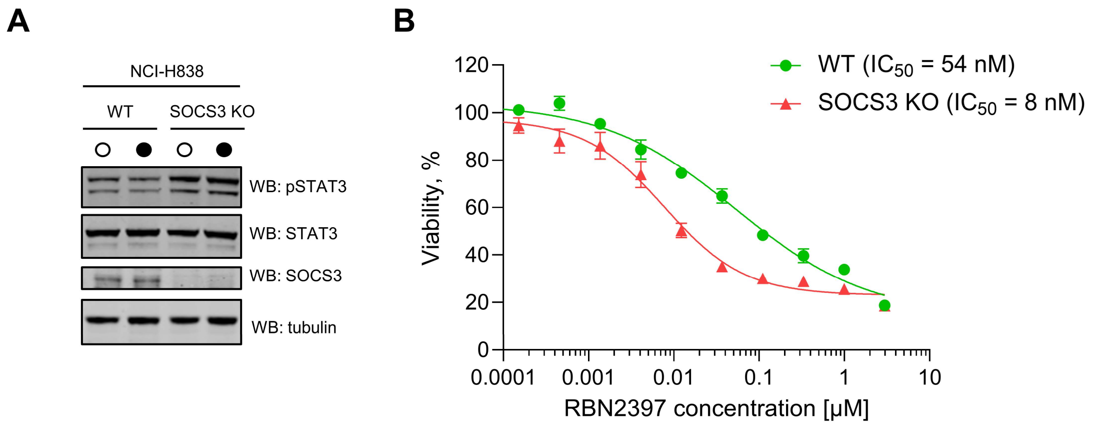

Fig. S17. SOCS3 knockout sensitizes H838 cells to PARP7 inhibition. **A**. Western blotting analysis of SOCS3 knockout H838 cells. RBN2397 treatment was performed at 1 µM for 24 h. **B**. CellTiter-Glo® assay on WT and SOCS3 knockout H838 cells. Cells were treated with a range of concentrations of RBN2397 for 6 days. Data are shown as mean ± s.e.m. of n = 2 biological replicates (with 2 technical replicates each).

Fig. 2F uncropped western blots

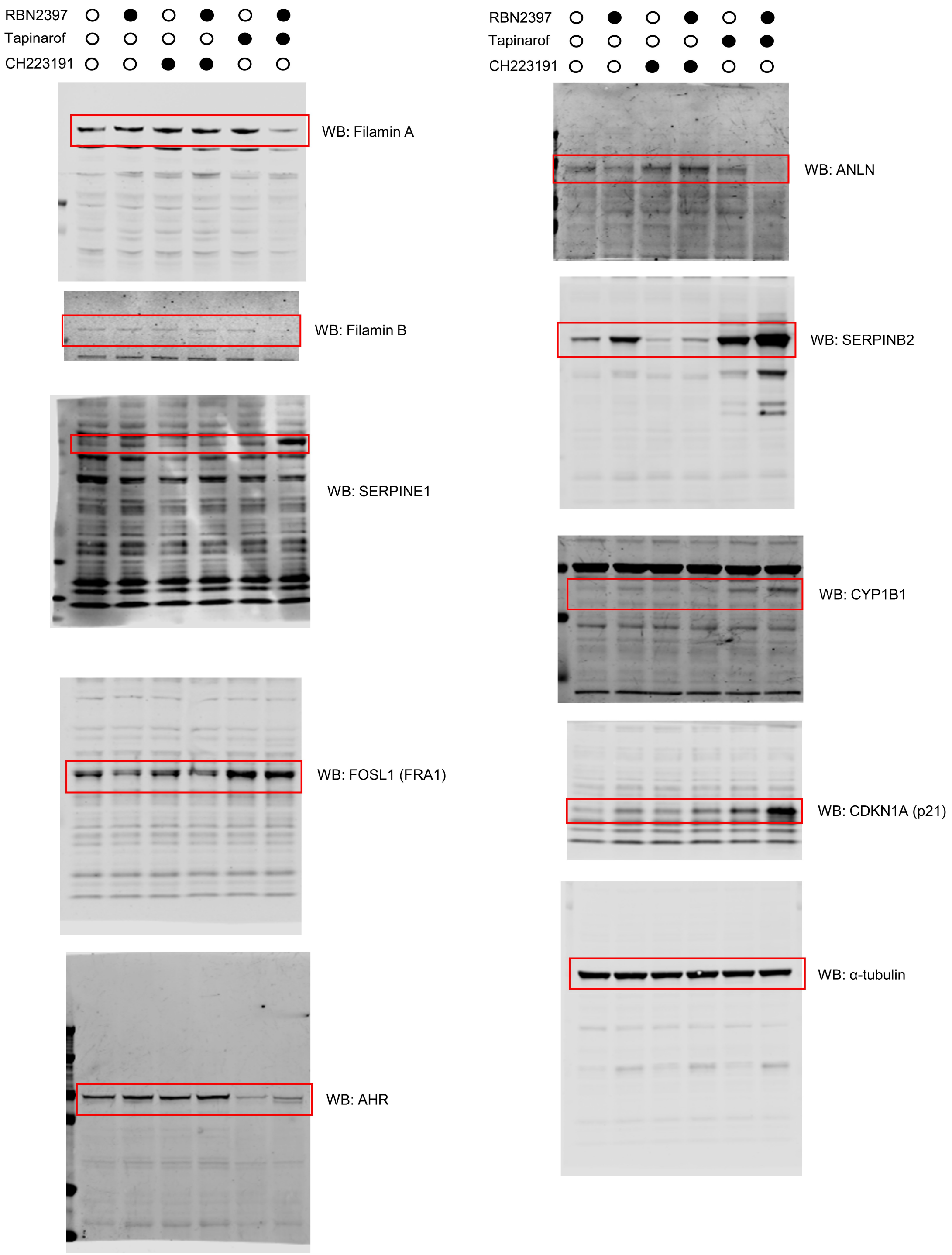

Fig. S18. Uncropped western blots for fig. 2F.

Fig. 3C uncropped western blots

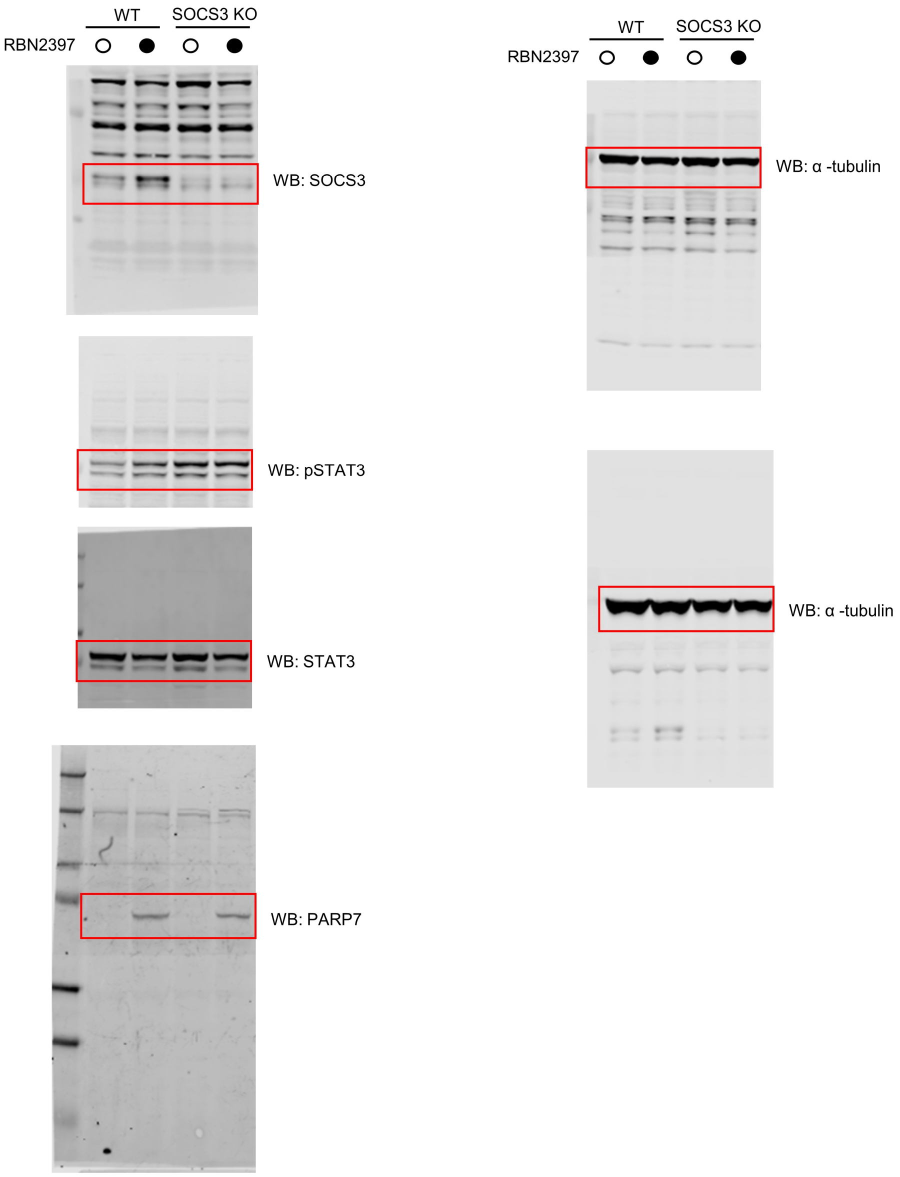

Fig. S19. Uncropped western blots for fig. 3C.

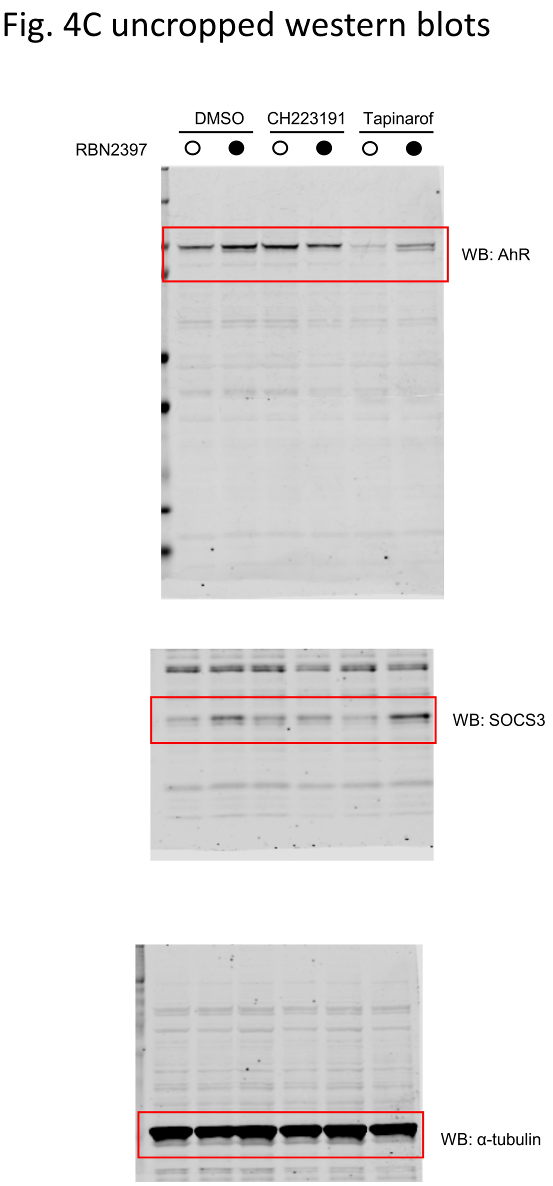

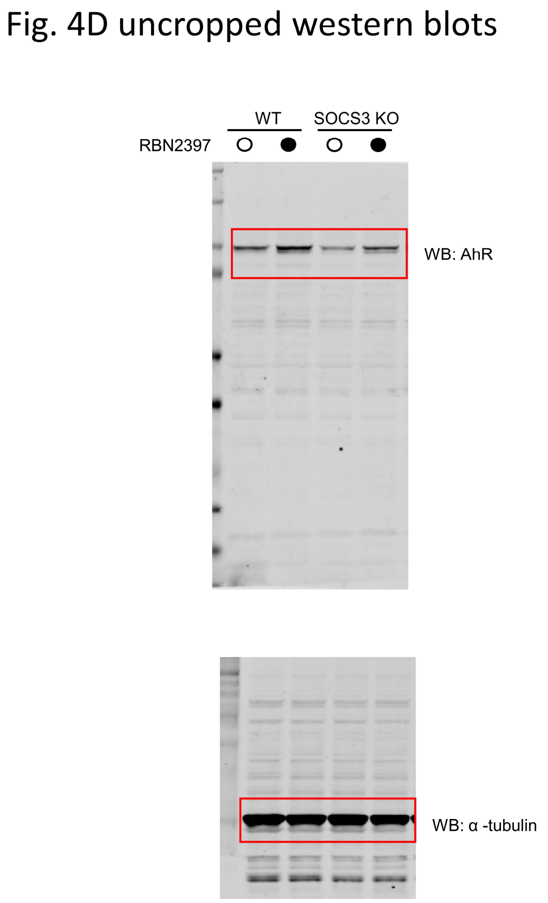

Fig. S20. Uncropped western blots for fig. 4C and 4D.

Supplementary Fig. 1 uncropped western blots

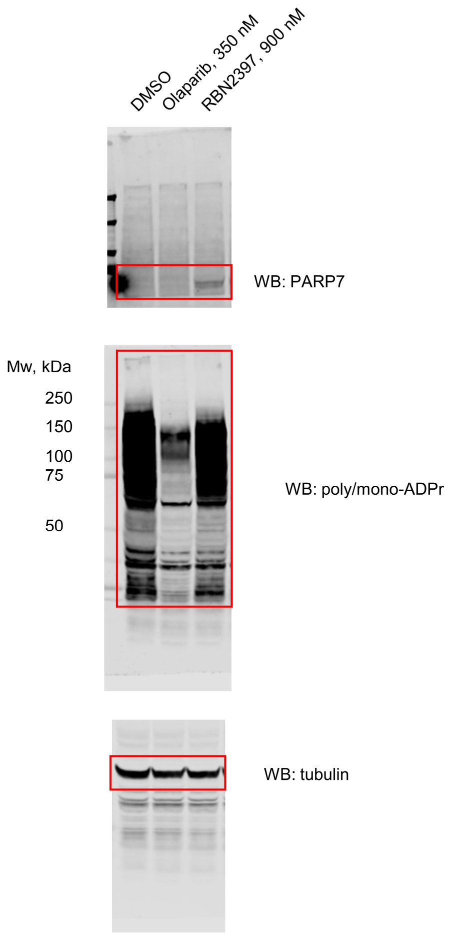

Fig. S21. Uncropped western blots for supplementary fig. 1.

Supplementary Fig. 11 uncropped western blots

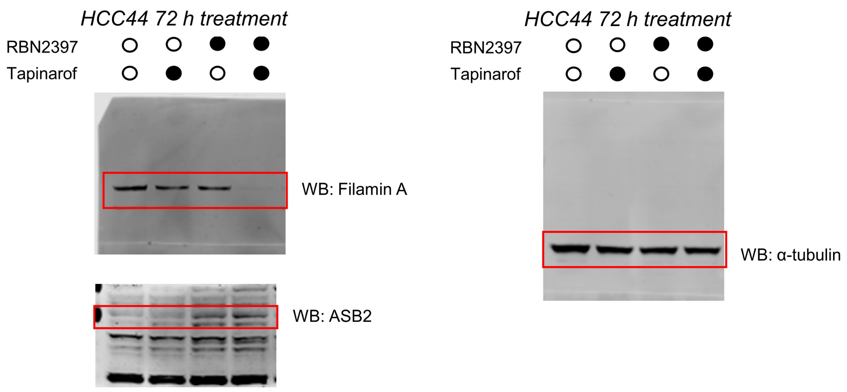

Supplementary Fig. 14A uncropped western blots

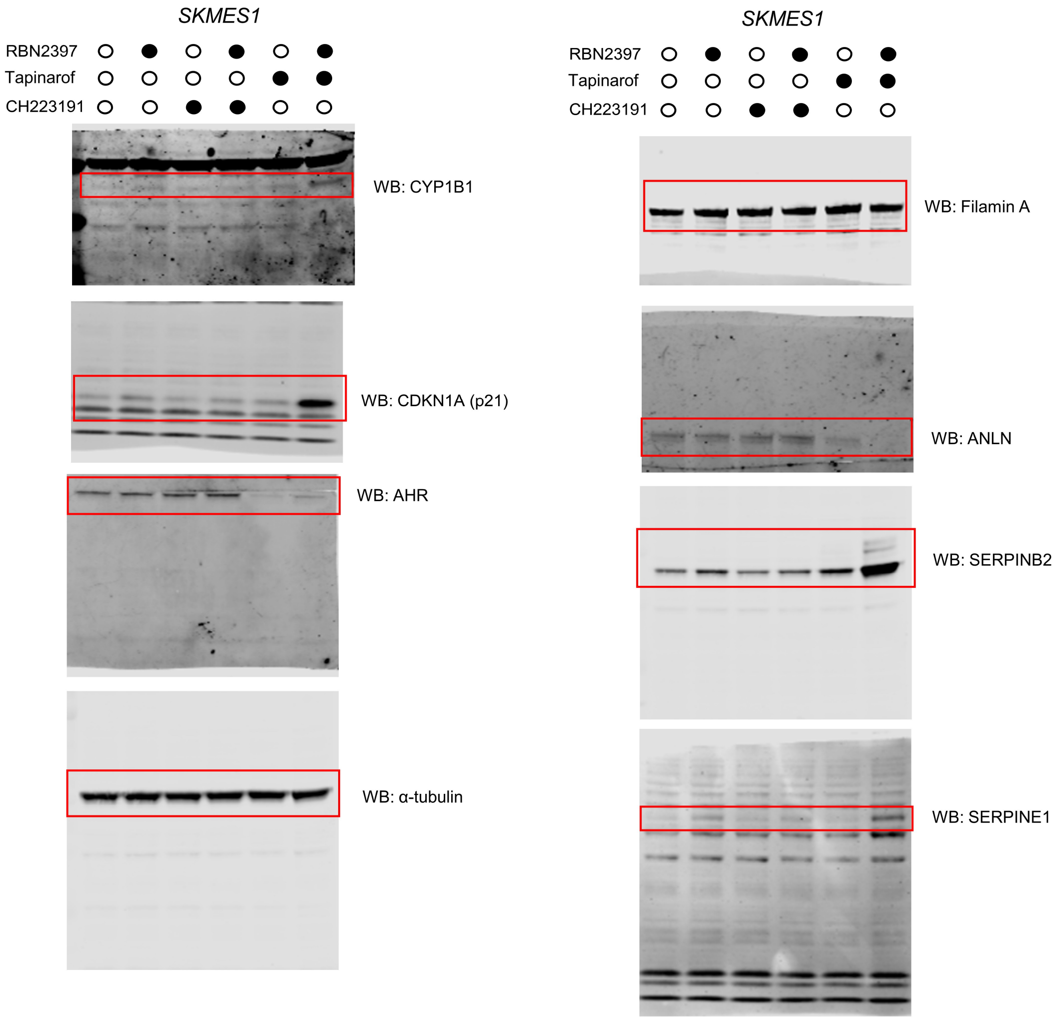

Fig. S22. Uncropped western blots for supplementary fig. 11 and 14A.

Supplementary Fig. 14B uncropped western blots

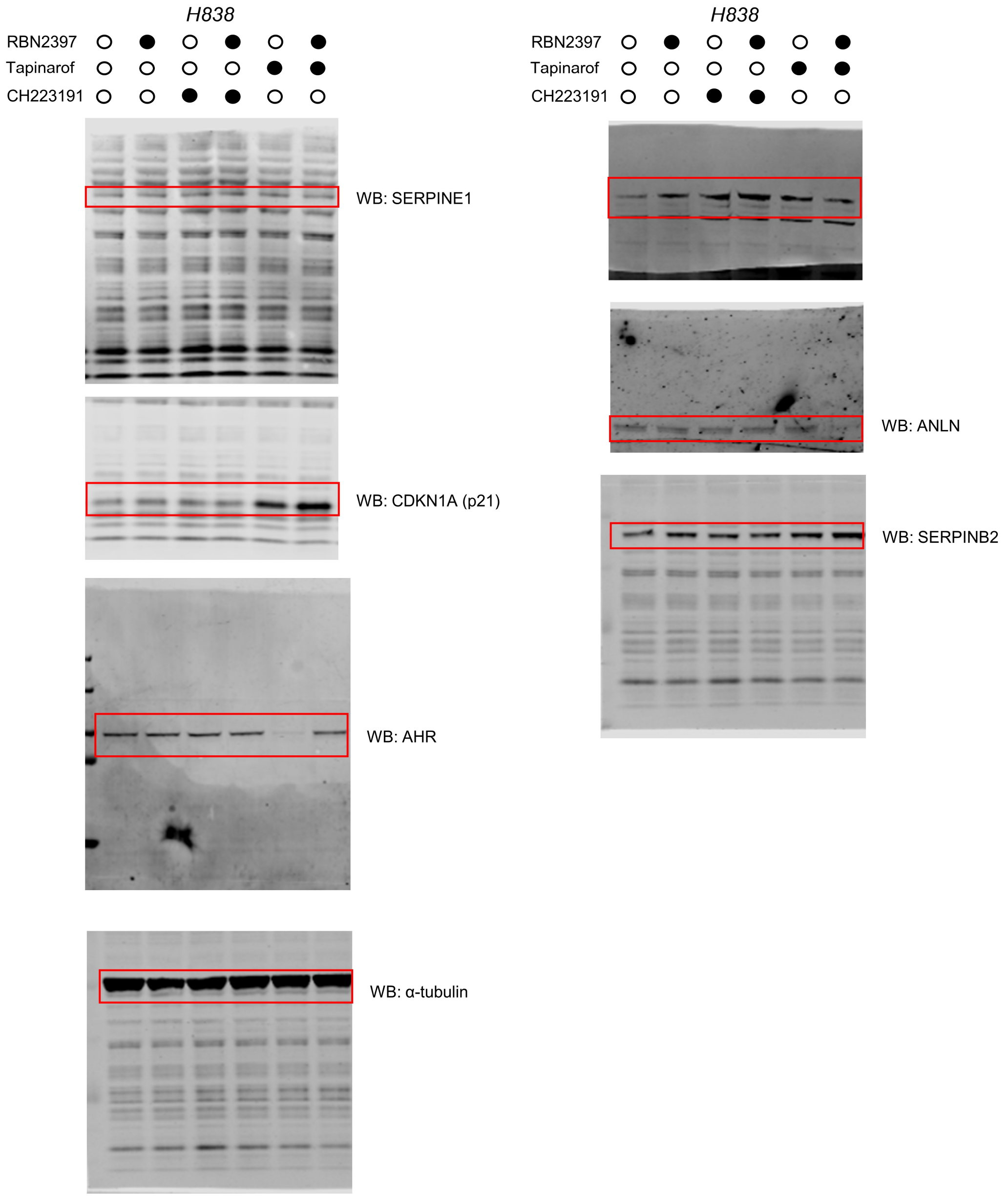

Fig. S23. Uncropped western blots for supplementary fig. 14B.

Supplementary Fig. 15 uncropped western blots

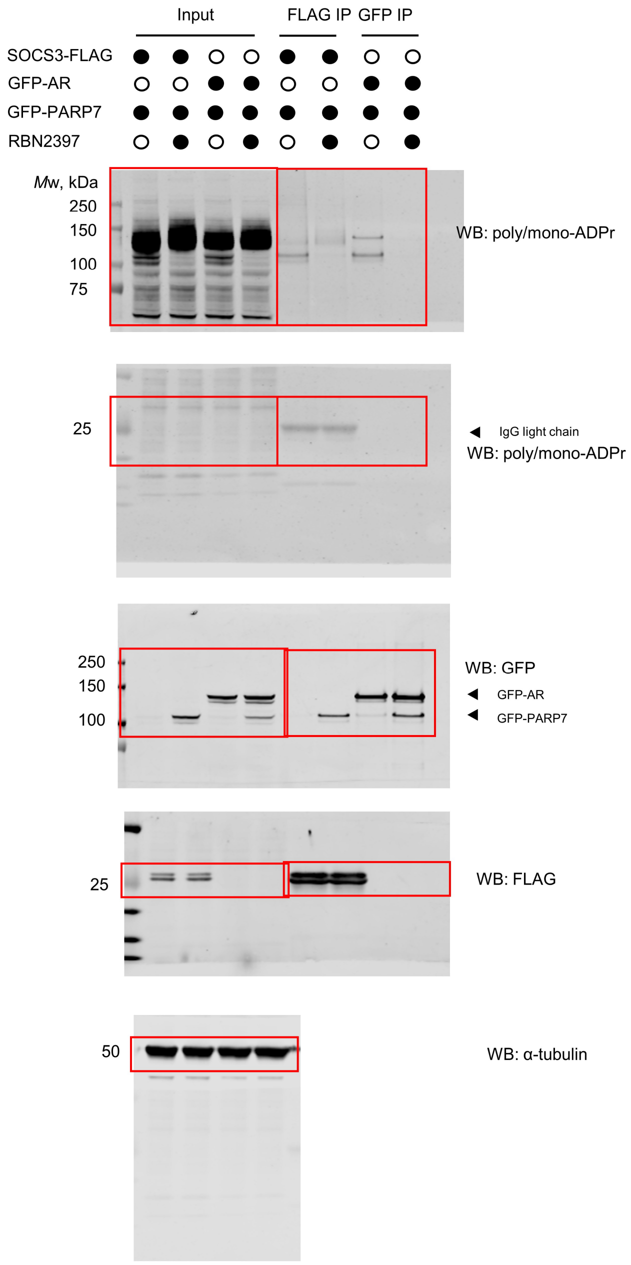

Fig. S24. Uncropped western blots for supplementary fig. 15.

Supplementary Fig. 17A uncropped western blots

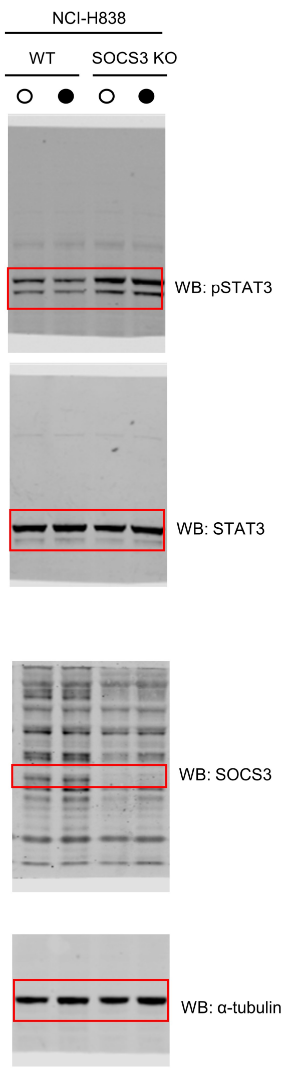

Fig. S25. Uncropped western blots for supplementary fig. 17A.

Dataset S1 (separate file). Supplementary table 1 (CRISPR screen).

Dataset S2 (separate file). Supplementary table 2 (CRISPR screen raw sgRNA count matrix).

Dataset S3 (separate file). Supplementary table 3 (CRISPR screen Mageck Bagel output).

Dataset S4 (separate file). Supplementary table 4 (sensitivity and resistance hits shared with other studies).

Dataset S5 (separate file). Supplementary table 5 (proteomics).

Dataset S6 (separate file). Supplementary table 6 (phosphoproteomics).

SI References

1. Hart T, Brown KR, Sircoulomb F, Rottapel R, Moffat J. Measuring error rates in genomic perturbation screens: gold standards for human functional genomics. Mol Syst Biol. 2014;10(7):733. Published 2014 Jul 1. doi:10.15252/msb.20145216
2. Behan FM, Iorio F, Picco G, et al. Prioritization of cancer therapeutic targets using CRISPR-Cas9 screens. Nature. 2019;568(7753):511-516. doi:10.1038/s41586-019-1103-9
3. Hart, T., & Moffat, J. (2016). BAGEL: a computational framework for identifying essential genes from pooled library screens. BMC bioinformatics, 17, 164. doi:10.1186/s12859-016-1015-8
